# Molecular mechanisms of plastic biodegradation by the fungus *Clonostachys rosea*

**DOI:** 10.1128/mbio.00335-25

**Published:** 2025-06-30

**Authors:** Victor Gambarini, Nikolai Pavlov, Paul Young, Stephanie Dawes, Arnaud Auffret, Joanne M. Kingsbury, Lloyd A. Donaldson, Dawn A. Smith, Louise Weaver, Olga Pantos, Kim M. Handley, Gavin Lear

**Affiliations:** 1 School of Biological Sciences, The University of Auckland 99026, Auckland, New Zealand; 2 The Institute of Environmental Science and Research 8480 , Christchurch, New Zealand; 3 Scion 6982 , Rotorua, New Zealand; Corporación CorpoGen, Bogotá D.C., Colombia

**Keywords:** *Clonostachys rosea*, cutinase, polycaprolactone, polyethylene terephthalate, plastic biodegradation

## Abstract

Microbial degradation can provide an avenue for the remediation of plastic pollution, contributing to the urgent environmental problem of global plastic waste. We demonstrate the degradation of polycaprolactone (PCL) by *Clonostachys rosea* and elucidate its underlying molecular mechanisms. We constructed the genome of this fungal strain and monitored changes in gene expression when exposed to PCL. Twelve genes linked to PCL degradation were found in the genome of *C. rosea*, and some of them were upregulated in the presence of the plastic, including genes coding for two cutinases. We heterologously expressed the enzymes coded by both genes and confirmed their activity against PCL polymers. We also demonstrate that one of the enzymes was active against polyethylene terephthalate polymers. Glucose inhibited the expression of both genes, completely halting the plastic biodegradation process, possibly serving as a preferred and readily metabolizable carbon source compared with PCL. We confirm the presence of key metabolic pathways linked to PCL degradation in *C. rosea*, including fatty acid degradation, providing further evidence of the mechanisms central to plastic biodegradation.

**IMPORTANCE:**

Plastic pollution is one of our most pressing environmental challenges, with billions of tons of plastic waste accumulating in our ecosystems. While recycling helps, it cannot fully address this crisis, making it crucial to find new solutions. Our study reveals how a common soil fungus, *Clonostachys rosea*, can break down certain plastics, specifically polycaprolactone and polyethylene terephthalate. We identified the exact genes and enzymes responsible for this ability and showed how different environmental conditions affect the fungus's plastic-degrading capabilities. Notably, we discovered that adding glucose completely stops the fungus from breaking down plastic, suggesting that careful control of growth conditions is essential for effective plastic degradation. These findings are significant because they provide a detailed blueprint for optimizing plastic biodegradation using fungi, potentially leading to more effective ways to tackle plastic pollution. This research represents a crucial step toward developing practical, environmentally friendly solutions for plastic waste management.

## INTRODUCTION

Plastic pollution is an urgent global environmental problem due to the extensive use and mismanagement of synthetic plastics and their persistence in the environment. Fish, seabirds, turtles, and marine mammals often mistake plastic debris for food, leading to ingestion (1–3), which causes gastrointestinal blockages or damages organs, for example, by plastic-induced fibrosis (4), in some cases resulting in starvation and death. Meanwhile, entanglement in plastic debris can cause strangulation, injuries, and hamper mobility, reducing survival rates (5, 6). Plastic pollution also poses significant risks to human health. Plastics can release toxic chemical additives, such as phthalates, bisphenol A, and polychlorinated biphenyls, which, if ingested, can cause endocrine disruption, reproductive problems, and have carcinogenic effects (7, 8).

Conventional management, such as recycling, remains insufficient to address the global plastic waste problem (9, 10). Consequently, there is a growing interest in alternative plastic treatment methods, including biodegradation. Fungi have emerged as potential candidates for plastic degradation due to their diverse enzymatic capabilities, including the enzymatic degradation of recalcitrant organic substances (11–14). Fungal enzyme classes, including laccases, peroxidases, esterases, lipases, proteases, and ureases, have shown remarkable efficacy in degrading various plastics under laboratory conditions (13, 14), predominantly associated with members of the Basidiomycetes (15, 16) and Ascomycetes (17–19).

Fungal laccases (20, 21) and peroxidases (22) have shown some potential in degrading polyethylene (PE) and polyvinyl chloride (PVC), two of the most commonly used plastics, whereas esterases, such as cutinases and lipases, are effective in degrading polyethylene terephthalate (PET) (23), polycaprolactone (PCL) (24, 25), and polyurethane (PUR) (26). Additionally, fungal proteases have shown potential for polylactic acid (PLA) degradation (27). The diverse fungal enzymes involved in plastic degradation highlight fungi’s versatility and ability to target different types of plastics.

Despite the progress in understanding fungal enzymatic systems related to plastic degradation, identifying new microorganisms capable of efficiently degrading plastics, as well as the genetic machinery by which degradation is mediated, is of great interest. *Clonostachys rosea* (phylum Ascomycota), a saprophytic filamentous fungus, has emerged as a potential candidate for plastic degradation (28, 29). Bainier (30) was the first to characterize *C. rosea*, previously known as *Gliocladium roseum*. Schroers et al. (31) discovered that the morphology, ecology, teleomorph, and DNA sequences from the 28S rRNA gene of *G. roseum* were very distinct from other *Gliocladium* species, so they reclassified *G. roseum* as *C. rosea*. It has been isolated from soil, plants (such as barley, onion, strawberry, rose, and cocoa), insects, nematodes, and even wine (32–34). This wide distribution and ability to colonise different environments highlight the ecological flexibility of *C. rosea* (35).

Understanding the biological characteristics of *C. rosea* is crucial for harnessing its potential applications, such as for biocontrol and biodegradation. Genomic studies provide insights into the biology of *C. rosea*. Its genome contains more than 14,000 annotated protein-coding genes (36, 37), including cell wall-degrading enzymes, polyketide synthases, ABC transporters, and monooxygenases (38). Two studies have isolated and characterized *C. rosea* strains that can degrade PCL and polyurethane (PUR). Barratt et al. (28) found that *C. rosea* was one of the key fungal strains responsible for the degradation of soil‐buried polyester PUR. Urbanek et al. (29) isolated *C. rosea* strain 16G from the Arctic, which exhibited the capacity to degrade 53% of PCL film within 30 days at 28°C; a remarkable degradation efficiency even observed at lower temperatures (20°C and 21°C). These findings highlight the potential of *C. rosea* to be an effective degrader of plastic waste. Yet, the biological attributes of *C. rosea* that enable it to degrade plastic remain unclear.

We investigated the plastic-degrading capabilities of a strain of *C. rosea*. This isolate is hereafter referred to as *C. rosea* PCL, in reference to its plastic-degrading capabilities. First, we explored the capacity of this strain to degrade an extended range of plastics. After confirming the strain’s ability to degrade PCL, we explored the genetic and enzymatic systems that contribute to the PCL-degrading capabilities of *C. rosea* under various environmental conditions. The findings from this study provide insights into the genetic mechanisms, metabolic pathways, and enzymes used for fungi to degrade plastics, as well as the environmental variables that influence the plastic degradation process.

## MATERIALS AND METHODS

### Plastic polymers

PCL and polystyrene (PS) were acquired from Sigma Aldrich (USA; products 440752 and 331651, respectively). Both PCL and PS were in pellet form and had average molecular weights of 14,000 and 35,000 g/mol, respectively. PLA pellets (Ingeo Biopolymer 3052D grade PLA) had an average molecular weight of 116,000 g/mol and were purchased from NatureWorks LLC, USA. Amorphous PET film (250 µM thick) was obtained from Goodfellow Cambridge Ltd (Huntingdon, UK) and cut into 2 mm × 5 mm pieces. PET microparticles were prepared from this film by milling under liquid nitrogen in a SPEX Sample Prep 6970 EFM Freezer Mill at 10 cycles/s for a total grinding time of 25 min. The microparticles were fractionated using an Octagon 200CL Sieve Shaker (Endecotts Ltd, UK) through a series of sieves of descending size pores of 500, 355, 125, and 63 µm (Endecotts Ltd). Microparticles sized between 125 and 355 µm were used for assays.

### Preparation of polymer emulsions

PE liquid emulsions (Aquacer 1063) were a kind donation from ResChem (New Zealand). To prepare PCL, PS, and PLA polymer emulsions, 6 g of the respective polymer was dissolved in 100 mL dichloromethane (DCM) (Merck, Germany) at room temperature for 48 h. The solution was mixed with 300 mL of ddH_2_O and 1.2 mL of dishwashing liquid (Ecostore, New Zealand) and sonicated for 5 min at 50% amplitude, with 1-s ON and OFF pulses, using a Q700 sonicator with a 1/2 in. diameter probe (Qsonica, Newtown, CT, USA). DCM was then evaporated by stirring at 50°C in a fume hood for 1 h. After DCM evaporation, the solution was cooled to room temperature.

### Fungal strain and genome characterization

The strain of *C. rosea* utilized in our study was isolated in Rotorua, New Zealand, and provided by the Scion Research Institute in Rotorua, New Zealand. To isolate DNA from *C. rosea*, we used a PowerSoil DNA Isolation Kit (MoBio Laboratories, Carlsbad, CA, USA). First, the fungal strain was grown on potato dextrose agar plates at 25°C for 5 days, and then approximately 200 mg of mycelium was scraped off the plate using a sterile loop and transferred to a 2 mL PowerBead tube. We followed the manufacturer’s instructions for DNA extraction, except for the mechanical lysis step, which was performed using a TissueLyzer II (Qiagen, Valencia, CA, USA) for 2 min at 30 Hz.

After extraction, the genomic DNA was cleaned and concentrated using a DNA Clean & Concentrator-5 Kit (Zymo Research, Irvine, CA, USA), following the manufacturer’s instructions, and the pellet was re-suspended in UltraPure DNase/Rnase-Free Distilled Water (20 µL; Cat No. 10977015; Invitrogen, Thermo Fisher Scientific, Waltham, MA, USA). The quality and quantity of the extracted genomic DNA were initially assessed using a Qubit 2.0 Fluorometer (Invitrogen, Carlsbad, CA, USA) and agarose gel electrophoresis to ensure the DNA was of high integrity and of sufficient concentration for DNA sequencing. Genomic DNA was stored for downstream applications at −80°C.

Whole-genome sequencing was performed by Auckland Genomics Ltd. (University of Auckland, NZ). Auckland Genomics performed library preparation and sequenced the sample DNA on an Illumina MiSeq platform using a 500-cycle MiSeq Nano 2 × 250 bp kit, as per the manufacturer’s protocols.

### Biodegradation assays on plastic-emulsion agar plates

M9 minimal agar was prepared by adding 11.28 g of M9, Minimal Salts, 5× concentrate (Merck, New Zealand; product M6030) to 1 L of distilled water and autoclaving for 20 min at 121°C. Following preparation, the medium was cooled to approximately 50°C. At this temperature, the previously prepared polymer emulsion was incorporated into the M9 medium at 50 mL of emulsion per litre of medium. The mixture was stirred thoroughly to ensure a uniform distribution of the polymer emulsion within the agar medium. Approximately 20 mL of this medium was dispensed into sterile 9 cm plastic petri dishes under aseptic conditions and allowed to solidify at room temperature.

*C. rosea* was inoculated onto the prepared agar plates' surface by transferring a fungal mycelium plug using a sterile cork borer. The plates were incubated at room temperature (18–24°C) and monitored daily, visually inspecting for microbial growth and any clear “halos” in the plastic emulsion, indicative of plastic degradation. We did not test PET degradation using emulsions because PET was insoluble in all the solvents we evaluated.

### PCL biodegradation assays incorporating varying carbon substrates

As other studies have found that adding supplementary carbon sources could improve the plastic degradation performance of some fungal species (39, 40), we tested the PCL degradation rates by *C. rosea* using different carbon sources. M9 minimal agar plates containing emulsified PCL were supplemented with either 1% (wt/vol) glucose, tryptic soya agar (Merck, Germany), citrate (Merck, Germany), potato starch (Merck, Germany), gelatine (Fisher Scientific, UK), fumarate (Merck, Germany), or cellulose (Sigma-Aldrich) to assess their potential influence on PCL biodegradation. Triplicate plates were inoculated with agar plugs of *C. rosea,* incubated at 25°C for 20 days, and monitored daily for signs of microbial growth and PCL degradation. Where observed, clearance zones were photographed to quantify the extent of biodegradation under the influence of each substrate. Images were processed using ImageJ software (NIH, USA), and the “measure” function was used to determine the diameter of the clear zones.

### RNA-Seq experiment

To identify the gene expression patterns associated with PCL degradation, we aimed to compare treatments in which the PCL degradation phenotype is either present or absent. Therefore, noting the inhibitory effect of glucose for PCL degradation by *C. rosea,* we cultured the organism under three media conditions: (i) PCL—M9 minimal agar supplemented with emulsified PCL; (ii) GLU—M9 minimal agar supplemented only with 1% (wt/vol) glucose (i.e., no PCL); (iii) GLUPCL—M9 minimal agar supplemented with both 1% (wt/vol) glucose and emulsified PCL. For each treatment (PCL, GLU, and GLUPCL), a plug of *C. rosea*, previously incubated under the same conditions, was transferred to Petri dishes to initiate the culture and incubated at 25°C for 6 days. Each of the three treatments was replicated five times, yielding 15 samples. Six days post-inoculation, the fungus was collected from each Petri dish using a sterile scalpel and transferred into separate micro-centrifuge tubes. These were immediately flash-frozen in liquid nitrogen to preserve the integrity of the cellular RNA.

### RNA extraction

The preserved *C. rosea* RNA was extracted using an RNeasy Mini Kit (Qiagen, Hilden, Germany) according to the manufacturer’s instructions, with some modifications. Before starting, β-mercaptoethanol (β-ME) was added to Buffer RLT at a ratio of 10 µL β-ME per 1 mL Buffer RLT. Buffer RPE was prepared by adding four volumes of ethanol (96–100%) to obtain a working solution.

To extract RNA, 60 mg of flash-frozen fungal biomass was weighed in 2 mL microfuge tubes and then subjected to cell lysis. The cell lysis process involved using a pre-chilled stainless steel adapter block, 2.8 mm stainless steel grinding balls, and a Geno/Grinder tissue homogenizer (SPEX SamplePrep, Metuchen, NJ, USA). Cell lysis was performed at 1,050 strokes/min for 1 min, followed by the addition of 700 µL Buffer RLT. The samples were ground twice at the same rate for 1 min each time and then ground once more for 2 min. After grinding, the samples were allowed to thaw at room temperature for 5 min. The RNA extraction process continued using a Qiagen RNeasy Mini Kit, following the manufacturer’s instructions, and eluting in RNAse-free water.

### RNA sequencing

Transcriptome sequencing was performed at Auckland Genomics, the University of Auckland, NZ. Before sequencing, the concentration and purity of the RNA were determined using a NanoDrop spectrophotometer (Thermo Fisher Scientific), and the integrity of the RNA was assessed with an Agilent 2100 Bioanalyzer (Agilent Technologies, Santa Clara, CA, USA). Library preparation for sequencing was conducted using the Illumina Stranded mRNA Prep Kit according to the manufacturer’s instructions (Illumina, Inc., San Diego, CA, USA).

Quality control checks were performed on the prepared library using a Bioanalyzer 2100 to confirm appropriate fragment size distribution, and the library was quantified using a Qubit 2.0 Fluorometer (Invitrogen, Carlsbad, CA, USA). Normalization and pooling of the libraries for simultaneous sequencing were done as required by the sequencing facility, and the final pool underwent another round of quality control. The RNA library was then sequenced on an Illumina HiSeq platform utilizing a 2 × 150 bp paired-end sequencing strategy.

### Film weight loss measurements

To confirm that the ability of *C. rosea* to degrade PCL is not limited to PCL emulsions, we fabricated PCL films by melting the polymer in a 70°C water bath and compressing it with glass, resulting in rectangular films measuring approximately 10 × 20 mm and weighing close to 0.1 g. Films were sterilized by exposure to 70% ethanol for 30 min. A biodegradation experiment was conducted to measure the weight loss of the polymer after incubation with *C. rosea*, using three distinct liquid media conditions: (i) 11.28 g/L of M9; (ii) 11.28 g/L of M9 and 4 g/L of yeast extract (yeast extract can frequently improve biodegradation rates [41]); (iii) 11.28 g/L of M9 and 4 g/L of inhibitory glucose (and no yeast extract). For each treatment, four replicates and two control flasks were prepared (i.e., containing only the media, with no fungal inoculation). In each 600 mL Erlenmeyer flask, 100 mL of the specific medium was introduced. An actively growing plug of *C. rosea* mycelium was added to each flask (except for the control samples), incubated for 180 days at 28°C, and agitated at 150 RPM.

Fungal mycelia were removed from the plastic films using sterile tweezers. The plastic films were then sterilized using a 70% ethanol solution for 10 min, followed by three rinses in sterile distilled water to remove residual ethanol and air-drying under a sterile laminar flow hood.

For scanning electron microscopy (SEM) imaging, sections of the plastic films were cut and attached to aluminum stubs using adhesive carbon tabs and sputter coated with 12 nm of chromium before examination using a JEOL 6700F field emission SEM at an accelerating voltage of 3 kV before examination at 1,000× magnification.

### Heterologous expression and protein purification

Two genes encoding the putative cutinases g9562 and g16887, upregulated in the presence of PCL, were synthesised and cloned into pET-28a(+)-TEV plasmids by GenScript Biotech PTE Ltd. (Singapore). The cutinase genes were cloned in the NdeI/EcoRI restriction enzyme sites of the plasmid, with the expressed protein containing an N-terminal hexa-histidine affinity tag and recombinant tobacco etch virus protease recognition site (rTEV). The plasmids (3 µL) were inserted via transformation into SHuffle T7 chemically competent *Escherichia coli* cells (New England Biolabs, Auckland, NZ) using a standard heat shock protocol. The transformed cells were plated on LB kanamycin plates and incubated for 24 h at 30°C. A single colony was cultured overnight in 10 mL of LB broth supplemented with kanamycin at 30°C and then transferred to 2 L baffled culture flasks containing 1 L of LB broth medium supplemented with kanamycin. The cultures were grown at 30°C with shaking to an OD_600nm_ of ~0.8 before induction with 0.3 mM isopropyl-β-d-1-thiogalactopyranoside (IPTG). The cultures were transferred to 18°C and incubated for 16 h before harvesting by centrifugation (15 min, 4,000 g). The cell pellet was re-suspended in 30 mL lysis buffer (50 mM Tris-Cl pH 8.0, 500 mM NaCl, 10 mM imidazole, and 0.5 mM TCEP) supplemented with complete protease inhibitor cocktail mini tablets (EDTA-free; Roche), transferred to 50 mL falcon tubes and stored at −20°C.

Cell pellets were lysed using a cell disruptor (Constant Cell Disruption Systems) at 124 kPa, and the lysate was clarified by centrifugation at 30,000 × *g* for 20 min at 4°C. The soluble portion was passed through a 5 mL IMAC column (HiTrap) pre-equilibrated with lysis buffer and bound protein washed with wash buffer (50 mM Tris-Cl pH 8.0, 300 mM NaCl, 20 mM imidazole). The recombinant protein was eluted in a linear gradient with elution buffer (50 mM Tris-HCl pH 8.0, 300 mM NaCl, and 500 mM imidazole) and analysed by SDS-PAGE to estimate purity.

The pure recombinant protein was pooled, and the buffer was exchanged into PBS. Briefly, the protein sample was concentrated to 1 mL in a 10-kDa-molecular-weight-cutoff protein concentrator (VivaScience), re-suspended with PBS buffer and reconcentrated. This was repeated twice to replace the elution buffer with PBS. Final protein concentrations were determined by measuring the absorbance of the samples at a wavelength of 280 nm using a NanoDrop spectrophotometer (Thermo Fisher Scientific).

### Enzyme kinetics

An enzyme kinetics experiment was conducted using the absorbance method published by Zhong-Johnson et al. (42). Briefly, 5 mg of PET powder and films were incubated in 2.5 mL of PBS buffer with varying enzyme concentrations: 50, 100, 150, and 200 nM. The samples were incubated for 5 days on a shaking rack at 100 rpm and a temperature of 30°C. Afterward, 1.5 µL from each sample was assessed using a NanoDrop at a wavelength of 245 nm. This specific wavelength was chosen based on the absorption characteristics of mono-(2-hydroxyethyl) terephthalic acid and terephthalic acid. Control samples, incubated with PBS buffer and PET but without enzyme, served as blanks for the NanoDrop readings. All measurements were taken in triplicate.

### Genome analysis

Before assembly, raw read quality was assessed using FastQC/0.11.7 (43). Adapters were removed using Trimmomatic /0.39 (44) with the options “PE” and “-baseout trimmomatic ILLUMINACLIP:1_trimmomatic/adapt.fa:2:30:10.” A fasta file, “adapt.fa,” containing two sequences, was supplied as input to Trimmomatic to remove sequences from the TruSeq Universal adapter (AATGATACGGCGACCACCGAGATCTACACTCTTTCCCTACACGACGCTCTTCCGATCT) and TruSeq Adapter Index 1 (GATCGGAAGAGCACACGTCTGAACTCCAGTCACATCACGATCTCGTATGCCGTCTTCTGCTTG). Low-quality bases were trimmed using “Sickle” (45) with the options “pe --qual-type sanger -l 70.”

The high-quality reads were assembled using SPAdes/3.13.1 (46) with a k-mer range of 21–127. To assess assembly completeness, we used BUSCO/3.0.2 (47) with the Pezizomycotina data set as a reference. The resulting assembly was further evaluated using QUAST/5.0.2 (48) to determine N50, L50, and genome size metrics.

To improve the accuracy of the assembly, we used BWA/0.7.17 (49) to align the raw reads to the assembly, followed by Pilon/1.23 (50) to perform error correction. The haplotigs in the assembly were identified and removed using “Purge Haplotigs” (51). Repetitive sequences in the genome were masked using “dustmasker” (52) to reduce their impact on gene prediction. Finally, we performed gene prediction using AUGUSTUS/3.3.2 with default parameters (53). The resulting genome assembly was used for further studies on this fungus.

The genome was annotated against several databases using BLAST (54) and HMMER3 (55), including KEGG (56), UniProt (57), Pfam (58), and TIGRfam (59). Signal peptides were annotated using SignalP 5.0 software (60). Additionally, all genome sequences were annotated using the InterProScan (61) tool with the -goterms flag, retrieving GO terms for each predicted protein sequence to investigate the biological significance of proteins of interest.

### RNA-seq analysis

The quality of the raw RNA-seq reads was checked using FastQC/0.11.9 (43) and MultiQC/1.13 (62). The raw reads were trimmed and filtered using fastp/0.23.2 (63) to remove low-quality bases and adapters. All parameters were set to their default value, but the following: “--trim_front1 9 --trim_front2 9.”

The trimmed reads were aligned to the reference genome using Bowtie 2/2.4.5 (64). The alignment file was sorted and indexed using SAMtools/1.10 (65). The featureCounts program (Subread/1.5.3) (66) was used to count the number of reads mapped to each gene. The count table generated by featureCounts was imported into the R/4.2.1 (67) environment for statistical and differential gene expression analysis. The DESeq2 package (68) was used to identify differentially expressed genes. The R-based topGO package was used to perform gene ontology (GO) enrichment analysis (69).

Transcripts with putative activity against PCL were identified by submitting the whole transcriptome to the webserver of the PlasticDB database (70), accessed on December 2024. The default values were used for the similarity search using the DIAMOND software version v2.0.6.144 (71) with an *e* value cutoff of 1e^−6^ and 30% identity on the amino acid level. AlphaFold2 (72) was used to predict protein structures through the ColabFold (73) platform with default values. Predicted structures were annotated based on their structure similarity against all the data deposited in the PDB database (74) using the web service PDBeFold (75).

### Statistical analysis

Statistical analyses were performed using R Statistical Software version 4.3.1 (67) and Python version 3.10.12 (76). A multivariate analysis assessed the impact of different experimental conditions on overall gene expression patterns. Principal component analysis (PCA) was conducted using the “prcomp” function in R, focusing on the top 500 most variable genes to retain maximum variance. Subsequently, we employed the “adonis2” function from the vegan version 2.6-4 (77) package in R to conduct a permutational multivariate analysis of variance (PERMANOVA). The analysis utilized a distance matrix based on PCA scores with the default Euclidean distance metric. A total of 999 permutations were executed to evaluate the significance of observed differences.

Statistical analyses were performed on two distinct data sets. The first data set assessed differences in gene expression values among the three treatment groups: GLU, PCL, and GLUPCL. The second data set investigated differences in clear zone areas among media types with various substrates. Initially, a one-way analysis of variance (ANOVA) was performed to assess overall differences among the treatment groups. Subsequently, Tukey’s honestly significant difference (HSD) test was applied as a post hoc analysis to identify specific treatment pairs exhibiting statistically significant mean differences. The ANOVA and Tukey’s HSD test were conducted using Python with the scipy.stats (version 1.10.1; Virtanen et al. [78]) and statsmodels (version 0.14.0; Seabold and Perktold [79]) libraries, respectively. The chosen significance level was *α* = 0.05 for both tests.

## RESULTS AND DISCUSSION

### Plastic degradation screening

The plastic-degrading abilities of the strain *C. rosea* PCL were investigated for four different plastic types: PCL, PLA, PS, and PE, emulsified in M9 minimal solid media. PET was not used due to its insolubility in all evaluated solvents. After 2 days of incubation, *C. rosea* exhibited a clear degradation halo on PCL-emulsified agar plates, indicating its ability to degrade PCL. However, there was no visible evidence of degradation on PLA, PS, and PE-emulsified agar plates after 21 days. No microorganism has yet been reported capable of degrading all four plastic types tested here. However, by searching the PlasticDB database, it is possible to find 36 microorganisms out of 633 in the database that can degrade both PCL and PLA (70).

**Fig 1:**
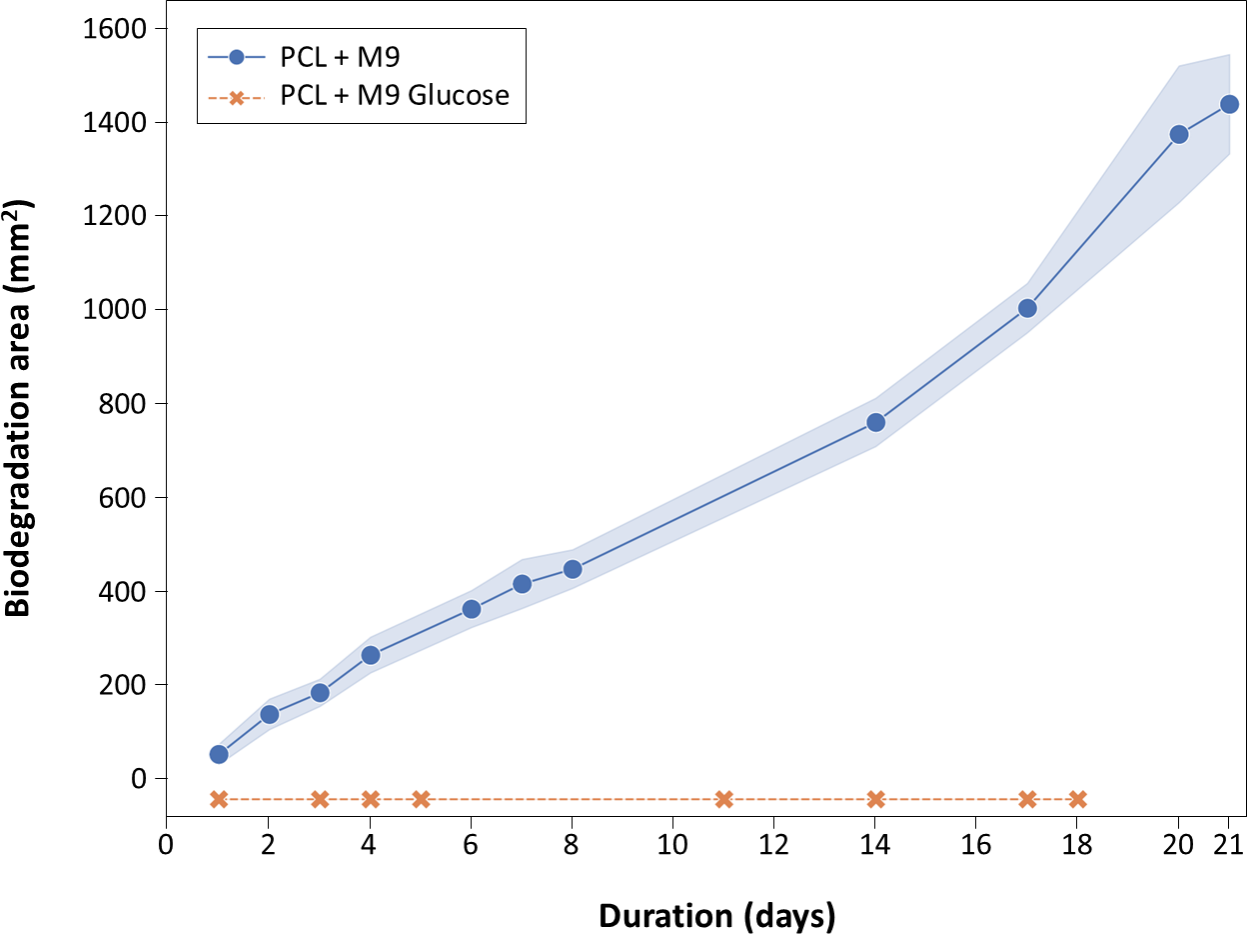
Ability of *Clonostachys rosea* to degrade a PCL emulsion in M9 media with and without glucose supplementation. Biodegradation was quantified by measuring the area of the clear halo formed around the microbial colonies. Each treatment was performed in triplicate. Shaded error bands represent standard deviation values. The ability of *C. rosea* to degrade PCL in the presence of cellulose, fumarate, gelatine, potato starch, citrate, and tryptic soy agar is shown in Fig. S1.

Following the identification of the PCL-degrading abilities of *C. rosea*, we investigated whether glucose supplementation affected PCL degradation. In biodegradation assays conducted over 18 days (Fig. 1), the M9 + PCL medium alone had a mean degradation area of 1,048 mm² (±53 StDev), demonstrating that *C. rosea* could grow using PCL as a sole carbon source. *C. rosea* did not grow on an agar-only medium, indicating it cannot use agar as a carbon source. Notably, PCL biodegradation was completely inhibited when glucose was added to the growth medium, with no evidence of biodegradation observed over 18 days. Additional substrate screening experiments examining the effects of various carbon sources on PCL degradation indicated that *C. rosea* with PCL-only medium may be carbon starved (Fig. S1).

The inhibition of PCL degradation in the presence of glucose may be attributed to glucose being a preferred and readily metabolizable carbon source compared with PCL. When glucose is available, the fungus may not produce the necessary enzymes for PCL degradation, leading to the complete inhibition of PCL degradation. This carbon catabolite repression is a common phenomenon and has also been reported in the literature for other carbon sources besides glucose (80–82).

**Fig 2:**
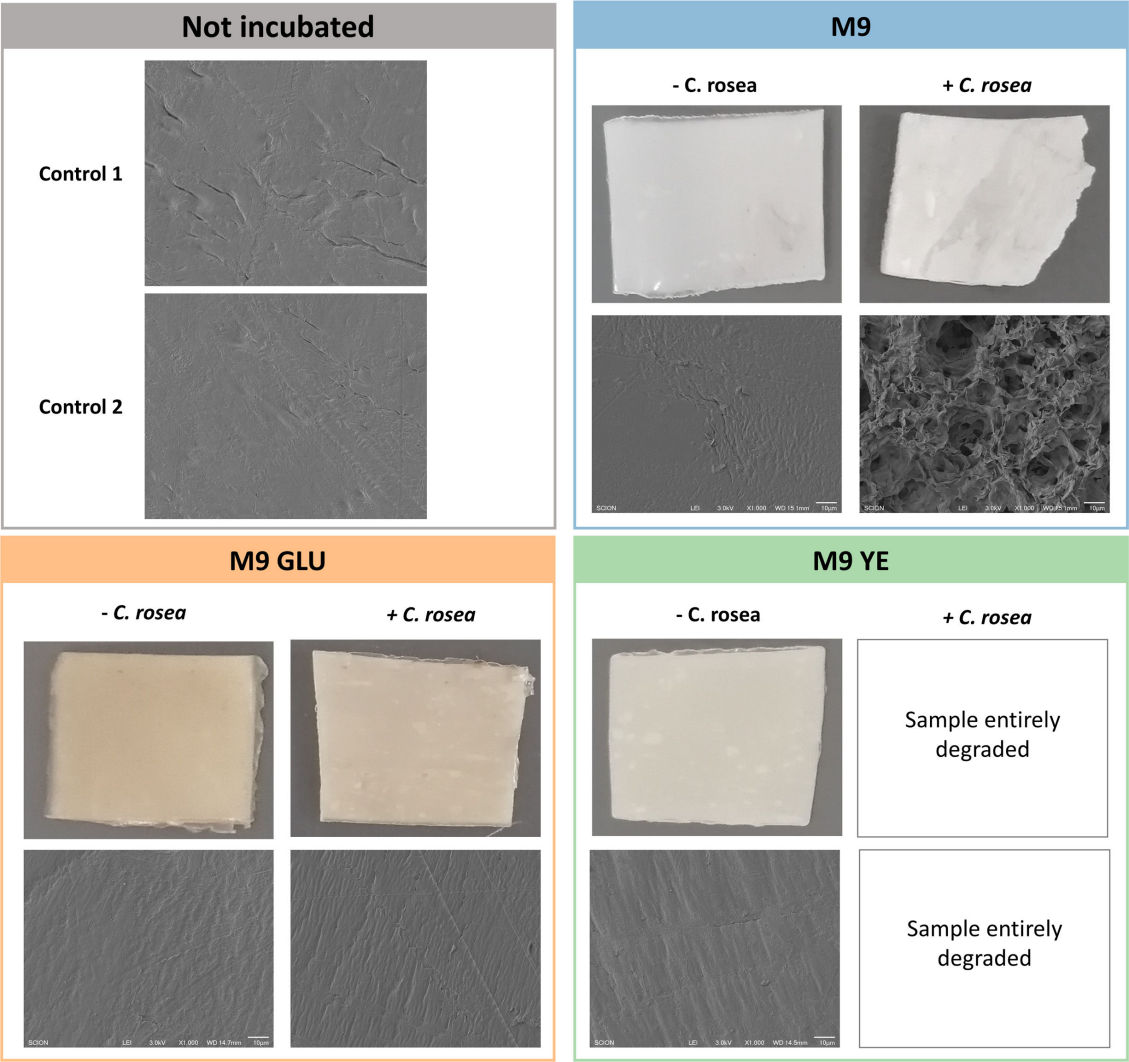
Biodegradation assay of PCL films by *Clonostachys rosea*. The fungus degraded PCL completely in the medium with yeast extract (YE), partially in the medium with M9 only, and did not degrade any PCL in the medium with glucose. The assay was conducted over a 180-day incubation period at 28°C on M9 liquid media supplemented with different substrates: M9, M9 + YE (4 g/L), and M9 + glucose (4 g/L), with all conditions replicated four times. The film weights were weighed before and after incubation to determine degradation percentages. Scanning electron microscopy (SEM) was performed on control and incubated samples at 1,000× magnification.

It is not uncommon to find examples in the literature where the plastic biodegradation of microorganisms is overstated (83), with microbes only degrading a small percentage of the plastic or degrading synthetically aged plastics. Therefore, we confirmed the degradation of solid PCL material, rather than emulsions by *C. rosea,* by conducting experiments using PCL films. Further, we confirmed the impact of glucose on PCL film degradation by *C. rosea,* using three distinct liquid media: M9, M9 with yeast extract (as a standard growth supplement), and M9 with glucose. Yeast extract was chosen as a more conventional growth medium supplement to ensure consistent microbial development, as it is frequently shown to improve biodegradation rates (41). After 180 days incubation at 28°C, *C. rosea* completely degraded (100%) PCL films in the M9 with yeast extract medium. In the M9 medium alone, 59.25% (SD: 7.10%) of the PCL films were degraded. In contrast, exposure to the medium supplemented with glucose resulted in only a 1.89% (SD: 0.58%) weight loss of the PCL film (Fig. 2).

Whether incubated or not, SEM images confirmed that control samples (samples not incubated or incubated without *C. rosea*) had a smooth smooth surface morphology. In the case of samples subjected to *C. rosea* incubation in the M9 glucose medium, no discernible differences were observed compared with the control samples, and signs of biodegradation were notably absent. In contrast, samples incubated with *C. rosea* on the M9 medium alone showcased a roughened surface characterized by cracks and voids. Samples incubated with *C. rosea* on the M9 yeast extract medium underwent complete degradation, precluding their inclusion in the SEM analysis.

### Genome analysis

**Fig 3:**
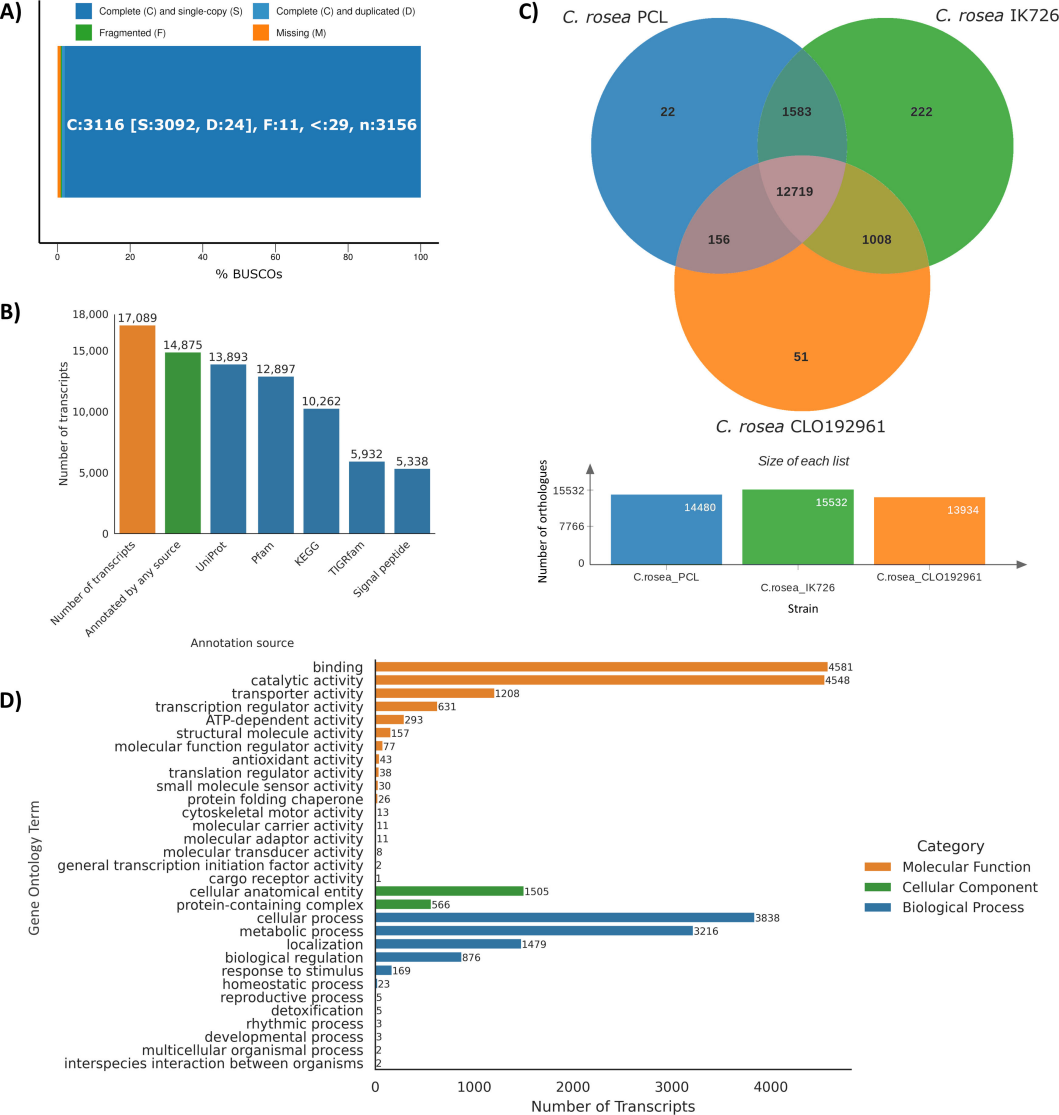
(A) BUSCO report of genome assembly completeness. (**B**) Number of annotated transcripts in the *C. rosea* genome for each annotation source. (**C**) Orthologous clusters shared between the sequenced genome from this study (*C. rosea* PCL) and two published *C. rosea* genomes. (**D**) Distribution of gene ontology (GO) terms annotated on the *C. rosea* genome.

To understand the genetic mechanisms involved in the ability of *C. rosea* to degrade PCL, we sequenced the fungus’ genome. The sequencing of the *C. rosea* genome resulted in 8,524,227 paired reads (Table S1). After quality control steps to remove low-quality reads, 98.75% of reads were retained. This retention rate suggests that reads used in consecutive analyses were of high quality. The size of *C. rosea’s* assembled genome is 56.8 Mbp, with 351 contigs and an average coverage of 31× (Table S2; Fig. S2). This estimation aligns well with previous reports of the *C. rosea* genome size, which ranges from 55.4 to 58.3 Mbp (36, 84). The N50 metric of 378.5 Kbp compares broadly with previously published values for *C. rosea* genomes of 569 Kbp (36) and 790 Kbp (37), and indicates a highly contiguous assembly. Furthermore, we analyzed the assembled genome to assess its content with respect to universal single-copy orthologs from the Pezizomycotina subphylum. This analysis verified that the assembled genome encompassed a significant portion of the anticipated universal single-copy orthologs. Specifically, 3,116 out of 3,156 orthologs were identified as present and complete (Fig. 3A). DNA extraction from fungal mycelia targeted the haploid life stage of *C. rosea* (85). This was evident in the BUSCO results, which showed abundant single-copy genes (3,092 genes) and few duplicates (24 genes). The BUSCO and QUAST analysis results indicated that the genome assembly did not contain any contamination, which was also confirmed by the NCBI FCS (Foreign Contamination Screen) pipeline (86).

A total of 17,089 genes were predicted in the genome of *C. rosea*. Overall, 2,214 genes (13%) were unannotated, with 14,875 genes (87%) annotated across all annotation sources (Fig. 3B). The annotation revealed 5,338 signal peptides involved in directing proteins to the correct location in the cell through translocation (87). Annotation with the UniProt and UniRef100 databases gave the highest number of annotations (13,893 each). The GO annotation of the *C. rosea* genome, which provides insights into the biological processes, molecular functions, and cellular components, suggests that, as most genomes (88), a significant portion of genes encode fundamental cellular activities like metabolic and cellular processes, as well as the spatial arrangement within the cell (Fig. 3D).

To compare the genome of the *C. rosea* strain described here (referred to as *C. rosea* PCL) to other published *C. rosea* genomes, that is, strains IK726 and CLO192961 (NCBI Genome Assembly accession numbers GCA_902827195.2 and GCA_902085965.1, respectively), we used the OrthoVenn3 tool (Fig. 3C). Out of the total clusters of orthologues identified in each strain, there were 14,480 in strain PCL, 15,532 in IK726, and 13,934 in CLO192961. In addition, 12,719 clusters were shared among all three strains, emphasizing these as core genomic elements underlying the biology of these strains. Interstrain comparative analysis revealed varying levels of shared orthologs between pairs of strains. There were 1,583 shared clusters between PCL and IK726, 156 between PCL and CLO192961, and 1,008 between CLO192961 and IK726. These pair-wise shared clusters indicate that strain PCL is more similar to strain IK726, making strain CLO192961 the most distinct among the analyzed genomes. Each strain also had unique ortholog clusters: PCL had 22 unique clusters, IK726 had 222, and CLO192961 had 51, illustrating genetic diversity among the strains. However, it is crucial to note that if the genomes are incomplete, then this observed diversity could be influenced by the incompleteness of the genomes.

### Gene expression profiling

**Fig 4:**
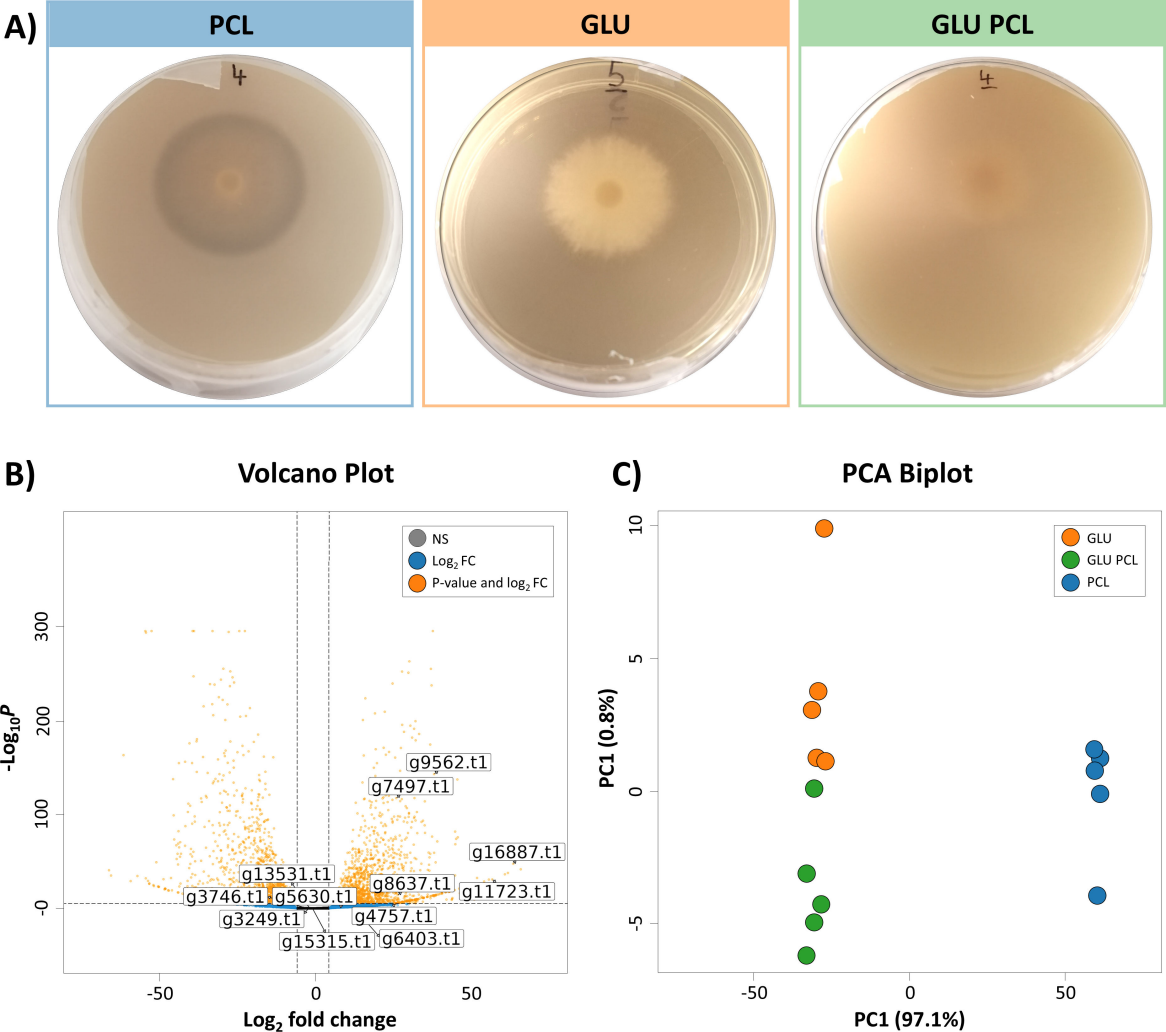
(A) *Clonostachys rosea* growing on the three media types used for the RNA-Seq experiment: (i) M9 agar with polycaprolactone (PCL), (ii) M9 agar with glucose (GLU), and (iii) M9 agar with both glucose and PCL (GLUPCL). All treatments had five replicates (*n* = 15). Clear zones can be observed on PCL-only plates but not on plates containing glucose. (**B**) Differentially expressed transcripts when comparing PCL versus GLU treatments, with cutoffs of FDR < 0.1 and absolute Log_2_FoldChange > 1. Positive Log_2_FoldChange values indicate the transcripts that were upregulated by the PCL treatment relative to the GLU treatment. Genes with labels were annotated as putatively encoding for PCL-degrading enzymes according to the PlasticDB database. (**C**) Principal component analysis (PCA) biplot of the expression values for the top 500 most variable genes. Significant differences in gene expression patterns among treatments were observed (R² =0.976, *P* < 0.001) based on PERMANOVA with 999 permutations using the Euclidean distance metric.

Next, we conducted an RNA-Seq experiment to identify putative genes and pathways for PCL degradation in *C. rosea* and the mechanisms for glucose-mediated inhibition of this process (Fig. 4A). We cultivated *C. rosea* for six days on the following solid media: M9 with PCL, M9 with glucose, and M9 with PCL and glucose. Next, RNA was extracted (mean RIN values of 7.04, SD: 1.46), and expression analysis was conducted. A total of 16,807 genes had mapped reads. No genes were notably downregulated in the GLUPCL medium (M9 medium with both glucose and emulsified PCL) compared with GLU medium control (M9 medium and glucose), while two genes were upregulated. Thus, adding emulsified PCL to the glucose medium did not significantly affect the gene expression profile of the fungus. In contrast, there were 1,835 significantly downregulated genes and 2,184 upregulated genes in the PCL medium (M9 medium with emulsified PCL) compared with the GLUPCL medium. Similarly, the comparison between PCL and GLU media showed 1783 significantly downregulated genes and 2228 upregulated genes in the PCL media (Fig. 4B), showing that the two treatments with glucose generated a very different gene expression profile compared to the PCL-only treatment.

A PCA Biplot showed that samples from the three treatments had compositionally distinct gene expression profiles (Fig. 4C). Samples with and without glucose separated principally across the *x*-axis, which accounts for a substantial 97.1% of the total data variance, further illustrating the influence of glucose on *C. rosea* gene expression. While GLU and GLUPCL samples clustered together on the *x*-axis, they separated along the *y*-axis. However, the *y*-axis explained only 0.8% of the total data variance, implying that the difference caused by PCL presence was small compared to the effect of glucose addition.

**Fig 5:**
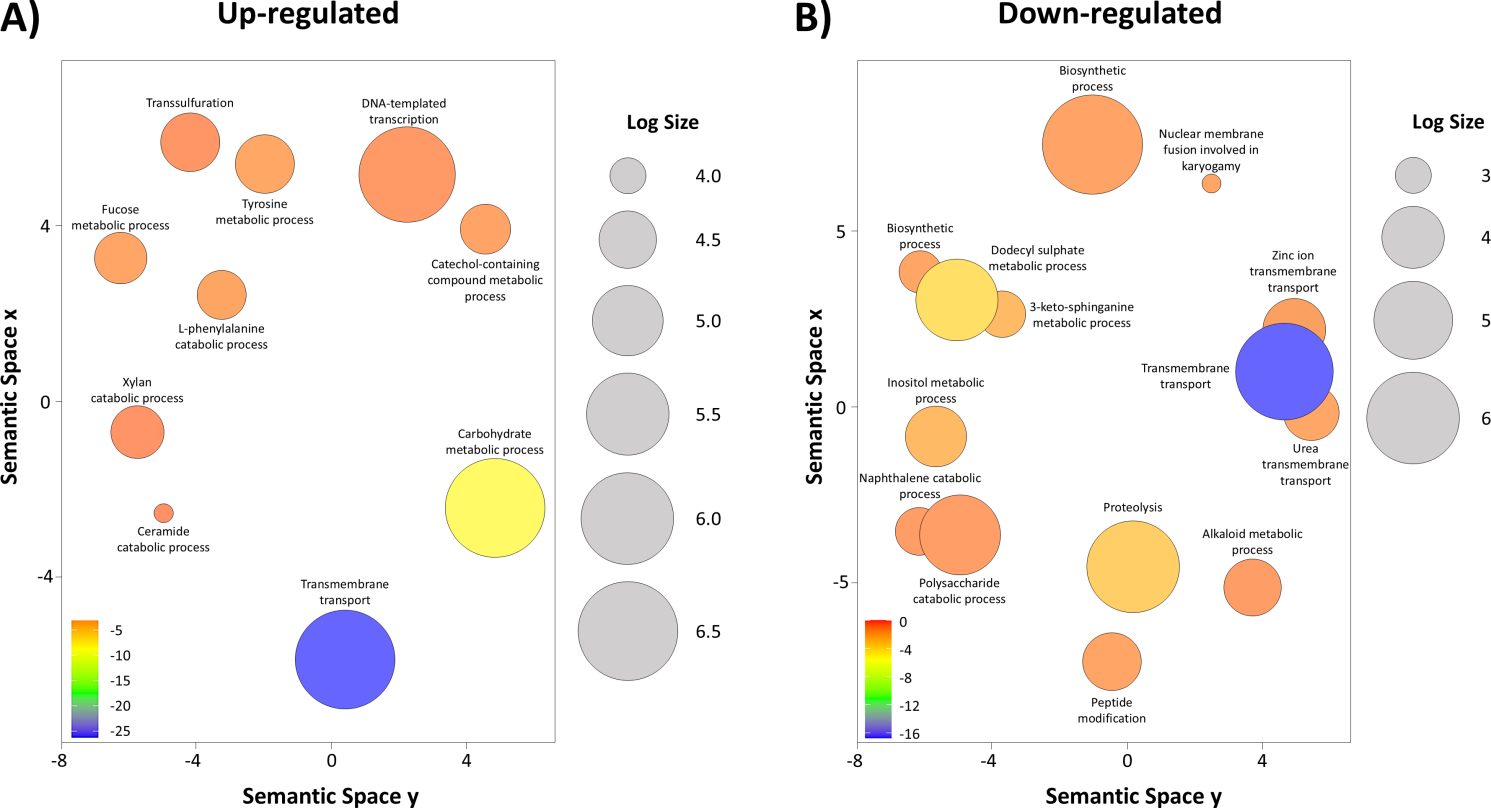
Gene ontology (GO) enrichment analyses summarised and visualised using REVIGO. Significantly enriched GO terms related to biological processes: (A) upregulated and (B) downregulated transcripts comparing PCL versus GLU-GLUPCL. GO terms are represented by circles and are clustered according to semantic similarities to other GO terms. Circle size is proportional to the frequency of the GO term, whereas color indicates the log_10_ *P* value for the enrichment derived from the topGO analysis.

As discussed earlier, *C. rosea* degradation of PCL was inhibited in the presence of glucose. Thus, biological processes upregulated in the PCL treatment compared with the GLU treatment may better elucidate the potential degradation pathways of PCL (Fig. 5A). Therefore, we performed a GO enrichment analysis and found the upregulation of several biological processes, including carbohydrate metabolic process, fucose metabolic process, and xylan catabolic process in the presence of PCL and absence of glucose. The observed upregulation of carbohydrate and fucose metabolic processes under PCL-only conditions might reflect a complex metabolic response, though its precise role remains speculative. One possibility is that, in the absence of preferred carbon sources like glucose, *C. rosea* could be activating these pathways to: (i) scavenge alternative carbon sources, (ii) support potential cell wall remodeling that might aid in enzyme secretion, or (iii) produce precursors that could feed into beta-oxidation pathways involved in processing PCL degradation products. These hypotheses suggest that *C. rosea* may possess metabolic flexibility that potentially allows it to cope with and possibly utilize PCL when more favorable substrates are absent.

We also found upregulation of genes involved in aromatic amino acid metabolism, such as l-phenylalanine and tyrosine, and catechol-containing compound metabolic process. Those upregulated biological processes may indicate that *C. rosea* may mobilize pathways to metabolise the aromatic components produced by the breakdown of the PCL polymer, such as ε-caprolactone. We also found several downregulated biological processes during PCL degradation, including polysaccharide and inositol catabolic processes, proteolysis, and fatty acid biosynthetic processes (Fig. 5B).

**Fig 6:**
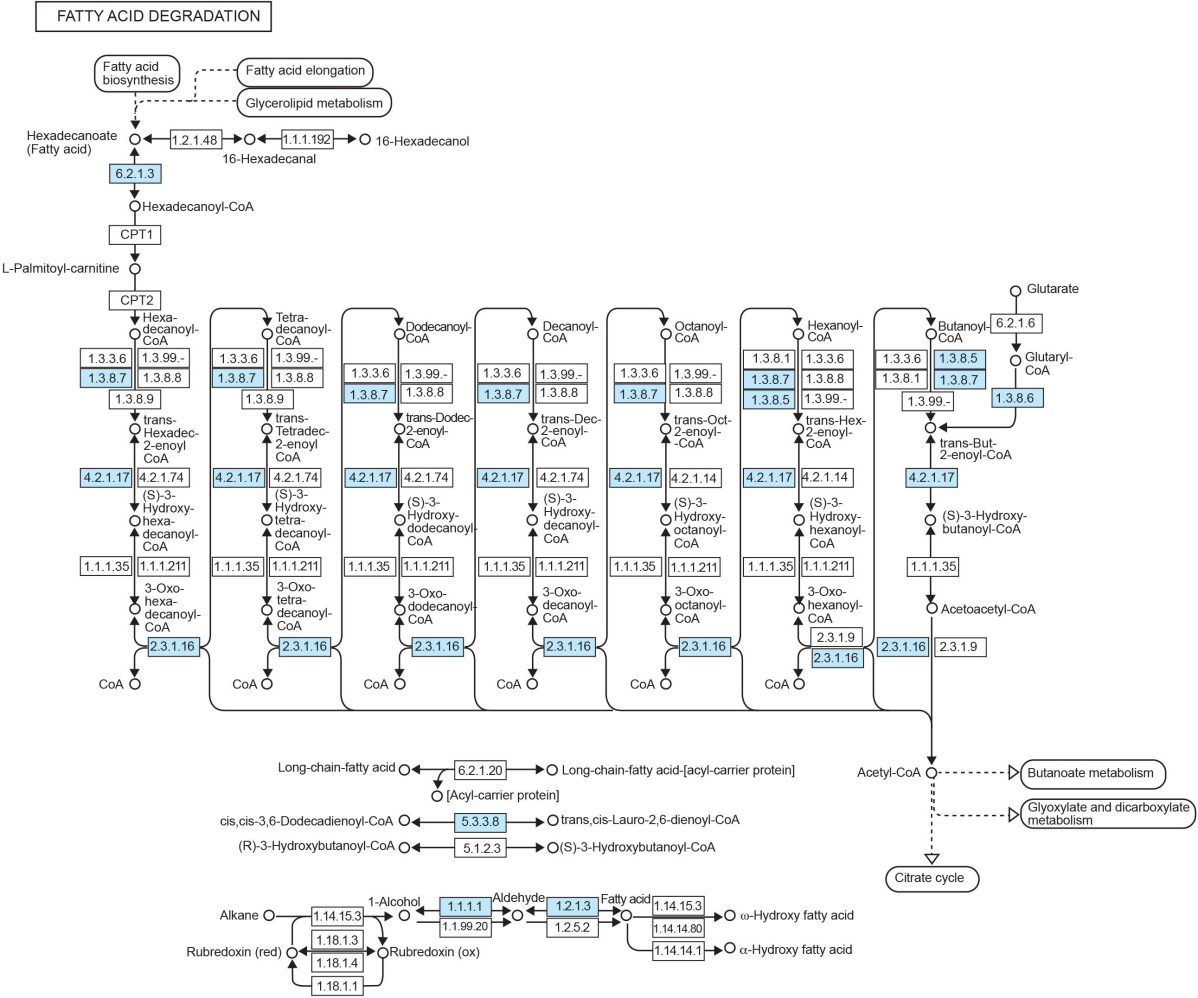
Upregulated expressed genes mapping to the fatty acid degradation pathway. Blue rectangles represent reactions upregulated in PCL compared to GLU and GLUPCL treatments, with cutoffs of FDR < 0.1 and absolute Log_2_FoldChange > 1.

The annotation of PCL-upregulated genes using the KEGG database reinforces insights gleaned from the GO analysis, identifying numerous genes involved in the fatty acid degradation pathway (Fig. 6). Fatty acid degradation, or beta-oxidation, involves breaking down long-chain fatty acids into acetyl-CoA (89). This versatile metabolite feeds into the tricarboxylic acid cycle, generating ATP and reducing equivalents (90). The upregulation of genes within this pathway in the PCL treatment suggests that *C. rosea* may exploit similar enzymatic machinery to metabolise the products generated during PCL degradation. This pathway has also been suggested to play a role in plastic degradation by other fungi (91). Simultaneously, the presence of genes from the fatty acid degradation pathway could indicate a metabolic adaptation for energy production under the PCL conditions. In the previous GO enrichment analysis, fatty acid biosynthetic processes were downregulated, suggesting that the degraded fatty acids likely originate from PCL degradation rather than produced by *C. rosea*. Fungi typically accumulate substantial quantities of lipids within both their hyphae and spores. These lipids are crucial as carbon and energy reservoirs, particularly during periods of starvation and spore germination (92–94). Therefore, with the potentially reduced availability of traditional energy sources such as polysaccharides, as suggested by the downregulation of polysaccharide catabolic processes in the GO analysis, *C. rosea* might try to compensate by upregulating fatty acid degradation of internal resources or PCL degradation products.

### Heterologous expression and enzyme kinetics

**Fig 7:**
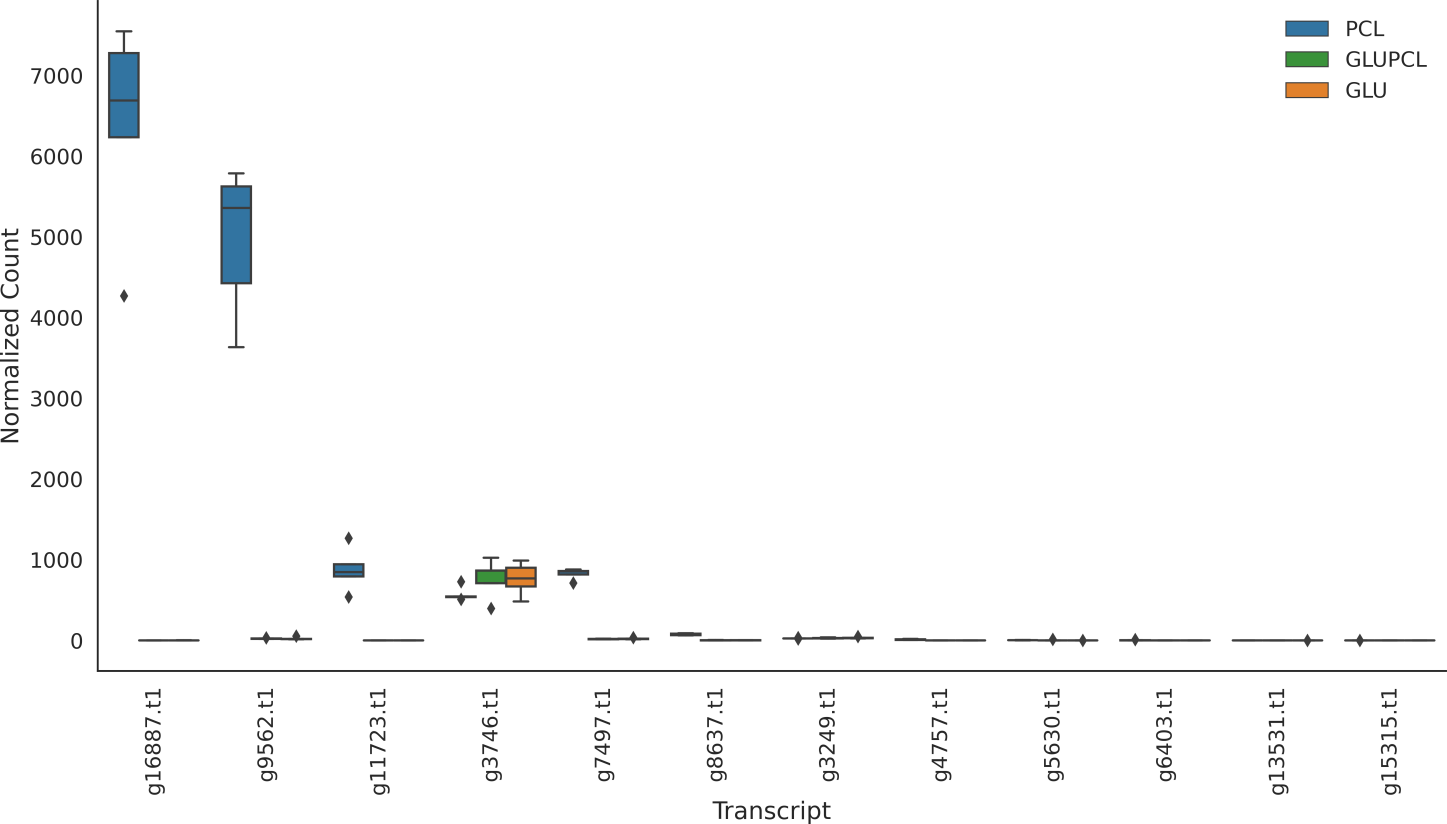
Expression of *Clonostachys rosea* transcripts annotated with putative PCL degradation activity by the PlasticDB tool. ANOVA (*F* = 14.96, *P* < 0.0001) indicated overall differences. Tukey’s HSD test for overall treatment effects revealed significant differences between [GLU and PCL], and [GLUPCL and PCL] treatments, but not between [GLU and GLUPCL] when considering all transcripts collectively. The box represents the range between the 25th percentile and the 75th percentile of the data. The line inside the box represents the median. The whiskers indicate the range of the data that are not considered outliers. Individual data points that fall outside the whiskers are outliers.

We identified 12 *C*. *rosea* genes as putatively coding for PCL-degrading enzymes by annotating all transcribed genes against PlasticDB, a database for genes reported to be involved in plastic biodegradation (70). Two transcribed genes, g16887.t1 and g9562.t1, stood out for being significantly differentially expressed and having high expression levels in the presence of PCL, while having an absence of expression in glucose treatments (Fig. 7; g16887.t1 log_2_FoldChange = 13.14; g9562 log_2_FoldChange = 11.96). These genes were, therefore, considered key candidates for PCL degradation. We also searched for these genes within the genomes of the *C. rosea* strains IK726 and CLO192961 using BLAST (54) with a cutoff *e* value of 1e−6 and a minimum percent identity of 30% at the amino acid level. Strain IK726 contained all 12 genes in its genome, while strain CLO192961 possessed 10.

The expressed g9562.t1 gene was identified by InterProScan as an alpha/beta-hydrolase. Based on PlasticDB, it was 66.8% identical to a cutinase from *Humicola insolens* (PlasticDB code: PLDB00121), demonstrated to degrade PET (95). Similarly, the expressed gene g16887.t1 was annotated by InterProScan as an alpha/beta-hydrolase. Based on PlasticDB, it was 68.3% identical to a cutinase from *Thermobifida fusca* (PlasticDB code: PLDB00073), another fungus reported to degrade PET (96). The action of cutinases, esterases, and hydrolases in plastic degradation is well documented, particularly due to their ability to hydrolyze ester bonds, a key structural feature of many plastics, including PCL (97, 98).

To confirm the ability of the enzymes coded by these two expressed genes to degrade PCL, we expressed the genes for these proteins in Shuffle T7 Competent *E. coli* cells and purified the resultant recombinant proteins via affinity chromatography. The molecular weight (Mw) for recombinant g9562.t1 was 20.0 and 34.1 kDa for g16887.t1. An aliquot (1 µL at a concentration of 2 µg/µL) of the purified enzymes was applied onto PCL-emulsified agar for 24 h at room temperature (18–24°C), and clear zones were observed for both enzymes, confirming their activity against PCL. The control with ddH_2_O showed no signs of degradation (Fig. S7).

**Fig 8:**
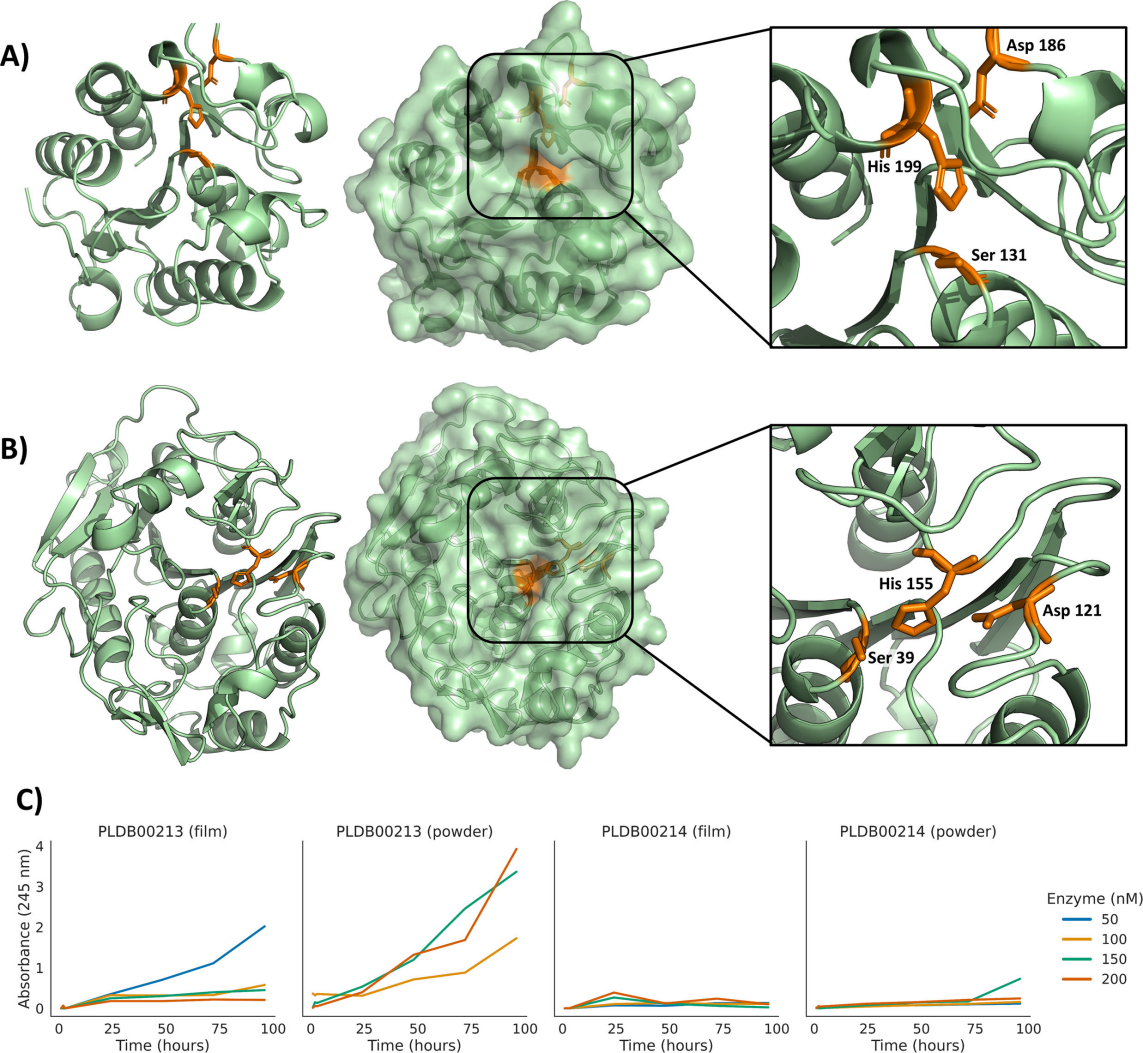
AlphaFold2 prediction of protein structures. Active site residues are shown in orange. (**A**) The cutinase PLDB00229 (g9562.t1) showed activity against PCL and PET. AlphaFold2 prediction scores: pTM = 0.85 and pLDDT = 91.4). (**B**) The putative cutinase PLDB00230 (g16887.t1) showed activity against PCL only. AlphaFold2 prediction scores: pTM = 0.92 and pLDDT = 94.9). (**C**) enzymatic degradation kinetics of PET powder and film using both purified enzymes. Degradation rates were determined using absorbance at representative enzyme concentrations; absorbance values are the mean of three readings per sample. Higher absorbance values indicate higher enzymatic activity against PET.

Various studies report that some enzymes capable of degrading PCL also possess the ability to degrade PET (23, 99, 100). The ability of these enzymes to degrade PET was therefore tested by an absorbance method (42). Only the enzyme expressed by the transcribed gene g9562.t1 exhibited concentration-dependent catalytic activity against PET, with higher enzyme concentrations leading to increased substrate degradation, effectively breaking down PET film and powder substrates over 5 days of incubation period (Fig. 8C). The enzymes coded by the transcribed genes g9562.t1 and g16887.t1 were deposited in the PlasticDB database as cutinases. g9562.t1 (accession: PLDB00229) was deposited with PCL and PET degradation activity, while g16887.t1 (accession: PLDB00230) was deposited with PCL degradation activity only.

We used the algorithm AlphaFold2 to predict the structure of enzymes PLDB00229 (g9562.t1) and PLDB00230 (g16887.t1). Using the predicted structure, we annotated both proteins using the PDBeFold web tool. PLDB00229 matched a cutinase from *H. insolens* (PDB: 4oyy; 65% sequence identity). Enzyme PLDB00229 active site is at residues SER 131, with HIS 199 and ASP 186 associated residues (Fig. 8A). It is in a long cleft and likely to accommodate longer polymer substrates. A putative mobile loop near the cleft may further expand the space available for substrate binding. Numerous similar structures of different cutinases share a similar substrate binding groove, indicating highly conserved functionality across species.

Enzyme PLDB00230 matched a polyhydroxybutyrate depolymerase from *Penicillium funiculosum* (PDB: 2d81; 69.2% identical) that was shown to degrade PHB and a trimer of R3HB (101). The enzyme PLDB00230 active site is predicted to be at residues SER 39, with HIS 155 and ASP 121 as associated residues (Fig. 8B). It is situated in a more constrained pocket when compared to the other enzymes. This smaller pocket may explain why PLDB00230 was only active against PCL, not PET. Furthermore, PLDB00230 and its *P. funiculosum* homolog (PDB: 2d81) share nearly identical residues lining the substrate binding pocket/groove. This structural similarity suggests that both enzymes likely exhibit comparable catalytic activity against polyesters.

### Conclusion

Our study demonstrates the plastic-degrading ability of *C. rosea*, particularly for PCL, while also highlighting the significant impact of glucose on this process. The fungus could use PCL as a sole carbon source, leading to substantial degradation in both emulsified and film forms. However, when glucose was added to the medium, PCL degradation was completely inhibited. This suggests that *C. rosea* prioritizes metabolizing glucose over producing enzymes required for PCL breakdown, a phenomenon consistent with carbon catabolite repression. In addition to PCL, we also demonstrate *C. rosea’s* ability to biodegrade PET, highlighting the influence of substrate composition on the biodegradation process. The biodegradation assays revealed that the carbon sources present significantly impact the degradation process, with potato starch providing the most favorable conditions out of those tested.

We demonstrated that the fungus underwent a significant shift in metabolic activity in the presence of PCL. Gene expression experiments highlighted the possible importance of the fatty-acid biodegradation pathway for plastic biodegradation. The analysis of upregulated transcripts against the PlasticDB database revealed two transcripts highly expressed under PCL conditions, suggesting their possible role in *C. rosea* PCL degradation phenotype. Both transcripts were heterologously expressed, and the produced enzymes exhibited activity against PCL, while one of the enzymes also demonstrated activity against PET polymers. The knowledge generated in this study can aid in developing more efficient microbiological strategies for plastic degradation and recycling by optimizing carbon sources, metabolic pathways, and enzyme activity on plastic substrates. Our findings emphasize the promising role of fungi in bioremediation and open doors to further research into enhancing the ability of fungi to degrade different types of plastics.

## Data Availability

The assembled genome, the raw genome Illumina paired-end sequences, and the raw RNA-Seq Illumina paired-end sequences have been submitted to the NCBI database, accession number PRJNA1017662, https://www.ncbi.nlm.nih.gov/sra/PRJNA1017662. The g9562.t1 and g16887.t1 protein sequences and their AlphaFold2 predictions were submitted to the PlasticDB database: https://plasticdb.org/proteins_229 and https://plasticdb.org/proteins_230, respectively. Some data and all the code used for bioinformatics and statistical analysis are organised in Jupyter notebooks deposited into GitHub: https://github.com/VictorGambarini/PlasticDegradersTree.

## SUPPLEMENTAL MATERIAL

Supplemental figures and tables mbio.00335-25-s0001.docx Figures S1 to S7; Tables S1 and S2.

ASM does not own the copyrights to Supplemental Material that may be linked to, or accessed through, an article. The authors have granted ASM a non-exclusive, world-wide license to publish the Supplemental Material files. Please contact the corresponding author directly for reuse.

## References

[1] Laist DW. 1987. Overview of the biological effects of lost and discarded plastic debris in the marine environment. Mar Pollut Bull 18:319–326. doi:10.1016/S0025-326X(87)80019-X

[2] Sigler M. 2014. The effects of plastic pollution on aquatic wildlife: current situations and future solutions. Water Air Soil Pollut 225:2184. doi:10.1007/s11270-014-2184-6

[3] Anastasopoulou A, Fortibuoni T. 2022. Impact of plastic pollution on marine life in the Mediterranean Sea, p 135–196. In Stock F, Reifferscheid G, Brennholt N, Kostianaia E (ed), Plastics in the aquatic environment - part I: current status and challenges. Springer International Publishing, Cham.

[4] Charlton-Howard HS, Bond AL, Rivers-Auty J, Lavers JL. 2023. “Plasticosis”: characterising macro- and microplastic-associated fibrosis in seabird tissues. J Hazard Mater 450:131090. doi:10.1016/j.jhazmat.2023.131090 36867907

[5] Egbeocha CO, Malek S, Emenike CU, Milow P. 2018. Feasting on microplastics: ingestion by and effects on marine organisms. Aquat Biol 27:93–106. doi:10.3354/ab00701

[6] Dar MA, Dhole NP, Pawar KD, Xie R, Shahnawaz M, Pandit RS, et al.. 2022. Ecotoxic effects of the plastic waste on marine fauna: an overview, p 287–300. In Shahnawaz M, Sangale MK, Daochen Z, Ade AB (ed), Impact of plastic waste on the marine biota. Springer Nature Singapore, Singapore.

[7] Kumar R, Manna C, Padha S, Verma A, Sharma P, Dhar A, Ghosh A, Bhattacharya P. 2022. Micro(nano)plastics pollution and human health: How plastics can induce carcinogenesis to humans? Chemosphere 298:134267. doi:10.1016/j.chemosphere.2022.134267 35301996

[8] Efferth T, Paul NW. 2017. Threats to human health by great ocean garbage patches. Lancet Planet Health 1:e301–e303. doi:10.1016/S2542-5196(17)30140-7 29628159

[9] Nikiema J, Asiedu Z. 2022. A review of the cost and effectiveness of solutions to address plastic pollution. Environ Sci Pollut Res Int 29:24547–24573. doi:10.1007/s11356-021-18038-5 35066854 PMC8783770

[10] Issifu I, Sumaila UR. 2020. A review of the production, recycling and management of marine plastic pollution. JMSE 8:945. doi:10.3390/jmse8110945

[11] Srikanth M, Sandeep TSRS, Sucharitha K, Godi S. 2022. Biodegradation of plastic polymers by fungi: a brief review. Bioresour Bioprocess 9:42. doi:10.1186/s40643-022-00532-4 38647755 PMC10991219

[12] Amobonye A, Bhagwat P, Singh S, Pillai S. 2021. Plastic biodegradation: frontline microbes and their enzymes. Sci Total Environ 759:143536. doi:10.1016/j.scitotenv.2020.143536 33190901

[13] Temporiti MEE, Nicola L, Nielsen E, Tosi S. 2022. Fungal enzymes involved in plastics biodegradation. Microorganisms 10:1180. doi:10.3390/microorganisms10061180 35744698 PMC9230134

[14] Sánchez C. 2020. Fungal potential for the degradation of petroleum-based polymers: an overview of macro- and microplastics biodegradation. Biotechnol Adv 40:107501. doi:10.1016/j.biotechadv.2019.107501 31870825

[15] Ali MI, Ahmed S, Javed I, Ali N, Atiq N, Hameed A, Robson G. 2014. Biodegradation of starch blended polyvinyl chloride films by isolated Phanerochaete chrysosporium PV1. Int J Environ Sci Technol 11:339–348. doi:10.1007/s13762-013-0220-5

[16] Huang M-H, Shih Y-P, Liu S-M. 2002. Biodegradation of polyvinyl alcohol by Phanerochaete chrysosporium after pretreatment with fenton’s reagent. J Environ Sci Health A 37:29–41. doi:10.1081/ESE-100108480 11846267

[17] Jung H-W, Yang M-K, Su R-C. 2018. Purification, characterization, and gene cloning of an Aspergillus fumigatus polyhydroxybutyrate depolymerase used for degradation of polyhydroxybutyrate, polyethylene succinate, and polybutylene succinate. Polym Degrad Stab 154:186–194. doi:10.1016/j.polymdegradstab.2018.06.002

[18] Crabbe JR, Campbell JR, Thompson L, Walz SL, Schultz WW. 1994. Biodegradation of a colloidal ester-based polyurethane by soil fungi. Int Biodeterior Biodegradation 33:103–113. doi:10.1016/0964-8305(94)90030-2

[19] Gambarini V, Pantos O, Kingsbury JM, Weaver L, Handley KM, Lear G. 2021. Phylogenetic distribution of plastic-degrading microorganisms. mSystems 6:01112–01120. doi:10.1128/mSystems.01112-20 PMC7820669 33468707

[20] Sumathi T, Viswanath B, Sri Lakshmi A, SaiGopal DVR. 2016. Production of laccase by Cochliobolus sp. isolated from plastic dumped soils and their ability to degrade low molecular weight PVC. Biochem Res Int 2016:9519527. doi:10.1155/2016/9519527 27293894 PMC4880699

[21] Kang BR, Kim SB, Song HA, Lee TK. 2019. Accelerating the biodegradation of high-density polyethylene (HDPE) using Bjerkandera adusta TBB-03 and lignocellulose substrates. Microorganisms 7:304. doi:10.3390/microorganisms7090304 31480475 PMC6780323

[22] Iiyoshi Y, Tsutsumi Y, Nishida T. 1998. Polyethylene degradation by lignin-degrading fungi and manganese peroxidase. J Wood Sci 44:222–229. doi:10.1007/BF00521967

[23] Dimarogona M, Nikolaivits E, Kanelli M, Christakopoulos P, Sandgren M, Topakas E. 2015. Structural and functional studies of a Fusarium oxysporum cutinase with polyethylene terephthalate modification potential. Biochim Biophys Acta 1850:2308–2317. doi:10.1016/j.bbagen.2015.08.009 26291558

[24] Murphy CA, Cameron JA, Huang SJ, Vinopal RT. 1996. Fusarium polycaprolactone depolymerase is cutinase. Appl Environ Microbiol 62:456–460. doi:10.1128/aem.62.2.456-460.1996 8593048 PMC167813

[25] Masaki K, Kamini NR, Ikeda H, Iefuji H. 2005. Cutinase-like enzyme from the yeast Cryptococcus sp. strain S-2 hydrolyzes polylactic acid and other biodegradable plastics. Appl Environ Microbiol 71:7548–7550. doi:10.1128/AEM.71.11.7548-7550.2005 16269800 PMC1287645

[26] Tokiwa Y, Calabia BP, Ugwu CU, Aiba S. 2009. Biodegradability of plastics. Int J Mol Sci 10:3722–3742. doi:10.3390/ijms10093722 19865515 PMC2769161

[27] Yamashita K, Kikkawa Y, Kurokawa K, Doi Y. 2005. Enzymatic degradation of poly(L-lactide) film by proteinase K: quartz crystal microbalance and atomic force microscopy study. Biomacromolecules 6:850–857. doi:10.1021/bm049395v 15762651

[28] Barratt SR, Ennos AR, Greenhalgh M, Robson GD, Handley PS. 2003. Fungi are the predominant micro-organisms responsible for degradation of soil-buried polyester polyurethane over a range of soil water holding capacities. J Appl Microbiol 95:78–85. doi:10.1046/j.1365-2672.2003.01961.x 12807456

[29] Urbanek AK, Rymowicz W, Strzelecki MC, Kociuba W, Franczak Ł, Mirończuk AM. 2017. Isolation and characterization of Arctic microorganisms decomposing bioplastics. AMB Express 7:148. doi:10.1186/s13568-017-0448-4 28697585 PMC5503855

[30] Bainier G. 1907. Gliocladium roseum sp. nov. et Cephalosporium acremonium (Corda). Bull Soc Mycol France 23:111–114.

[31] Schroers H-J, Samuels GJ, Seifert KA, Gams W. 2019. Classification of the mycoparasite Gliocladium roseum in Clonostachys as C. rosea, its relationship to Bionectria ochroleuca, and notes on other Gliocladium-like fungi. Mycologia 91:365–385. doi:10.1080/00275514.1999.12061028

[32] Krauss U, Martínez A, Hidalgo E, ten Hoopen M, Arroyo C. 2002. Two-step liquid/solid state scaled-up production of Clonostachys rosea. Mycol Res 106:1449–1454. doi:10.1017/S0953756202006822

[33] Mamarabadi M, Jensen B, Jensen DF, Lübeck M. 2008. Real-time RT-PCR expression analysis of chitinase and endoglucanase genes in the three-way interaction between the biocontrol strain Clonostachys rosea IK726, Botrytis cinerea and strawberry. FEMS Microbiol Lett 285:101–110. doi:10.1111/j.1574-6968.2008.01228.x 18557783

[34] Costa LB, Rangel DEN, Morandi MAB, Bettiol W. 2012. Impact of UV-B radiation on Clonostachys rosea germination and growth. World J Microbiol Biotechnol 28:2497–2504. doi:10.1007/s11274-012-1057-7 22806155

[35] Sun ZB, Li SD, Ren Q, Xu JL, Lu X, Sun MH. 2020. Biology and applications of Clonostachys rosea. J Appl Microbiol 129:486–495. doi:10.1111/jam.14625 32115828

[36] Karlsson M, Durling MB, Choi J, Kosawang C, Lackner G, Tzelepis GD, Nygren K, Dubey MK, Kamou N, Levasseur A, Zapparata A, Wang J, Amby DB, Jensen B, Sarrocco S, Panteris E, Lagopodi AL, Pöggeler S, Vannacci G, Collinge DB, Hoffmeister D, Henrissat B, Lee Y-H, Jensen DF. 2015. Insights on the evolution of mycoparasitism from the genome of Clonostachys rosea. Genome Biol Evol 7:465–480. doi:10.1093/gbe/evu292 25575496 PMC4350171

[37] Sun Z-B, Sun M-H, Li S-D. 2015. Identification of mycoparasitism-related genes in Clonostachys rosea 67-1 active against Sclerotinia sclerotiorum. Sci Rep 5:18169. doi:10.1038/srep18169 26657839 PMC4677357

[38] Qualhato TF, Lopes FAC, Steindorff AS, Brandão RS, Jesuino RSA, Ulhoa CJ. 2013. Mycoparasitism studies of Trichoderma species against three phytopathogenic fungi: evaluation of antagonism and hydrolytic enzyme production. Biotechnol Lett 35:1461–1468. doi:10.1007/s10529-013-1225-3 23690037

[39] Jarerat A, Tokiwa Y. 2001. Degradation of Poly(L-lactide) by a fungus. Macromol Biosci 1:136–140. doi:10.1002/1616-5195(20010601)1:4<136::AID-MABI136>3.0.CO;2-3

[40] Antipova TV, Zhelifonova VP, Zaitsev KV, Nedorezova PM, Aladyshev AM, Klyamkina AN, Kostyuk SV, Danilogorskaya AA, Kozlovsky AG. 2018. Biodegradation of poly-ε-caprolactones and poly-l-lactides by fungi. J Polym Environ 26:4350–4359. doi:10.1007/s10924-018-1307-3

[41] Oliveira J, Almeida PL, Sobral RG, Lourenço ND, Gaudêncio SP. 2022. Marine-derived actinomycetes: biodegradation of plastics and formation of PHA bioplastics-A circular bioeconomy approach. Mar Drugs 20:760. doi:10.3390/md20120760 36547907 PMC9783806

[42] Zhong-Johnson EZL, Voigt CA, Sinskey AJ. 2021. An absorbance method for analysis of enzymatic degradation kinetics of poly(ethylene terephthalate) films. Sci Rep 11:928. doi:10.1038/s41598-020-79031-5 33441590 PMC7806724

[43] Andrews S. 2010. FastQC: a quality control tool for high throughput sequence data. Available from: https://www.bioinformatics.babraham.ac.uk/projects/fastqc

[44] Bolger AM, Lohse M, Usadel B. 2014. Trimmomatic: a flexible trimmer for Illumina sequence data. Bioinformatics 30:2114–2120. doi:10.1093/bioinformatics/btu170 24695404 PMC4103590

[45] Joshi N, Fass J. 2011. Sickle: a sliding-window, adaptive, quality-based trimming tool for fastQ files. Available from: https://github.com/najoshi/sickle

[46] Bankevich A, Nurk S, Antipov D, Gurevich AA, Dvorkin M, Kulikov AS, Lesin VM, Nikolenko SI, Pham S, Prjibelski AD, Pyshkin AV, Sirotkin AV, Vyahhi N, Tesler G, Alekseyev MA, Pevzner PA. 2012. SPAdes: a new genome assembly algorithm and its applications to single-cell sequencing. J Comput Biol 19:455–477. doi:10.1089/cmb.2012.0021 22506599 PMC3342519

[47] Simão FA, Waterhouse RM, Ioannidis P, Kriventseva EV, Zdobnov EM. 2015. BUSCO: assessing genome assembly and annotation completeness with single-copy orthologs. Bioinformatics 31:3210–3212. doi:10.1093/bioinformatics/btv351 26059717

[48] Gurevich A, Saveliev V, Vyahhi N, Tesler G. 2013. QUAST: quality assessment tool for genome assemblies. Bioinformatics 29:1072–1075. doi:10.1093/bioinformatics/btt086 23422339 PMC3624806

[49] Li H, Durbin R. 2009. Fast and accurate short read alignment with Burrows-Wheeler transform. Bioinformatics 25:1754–1760. doi:10.1093/bioinformatics/btp324 19451168 PMC2705234

[50] Walker BJ, Abeel T, Shea T, Priest M, Abouelliel A, Sakthikumar S, Cuomo CA, Zeng Q, Wortman J, Young SK, Earl AM. 2014. Pilon: an integrated tool for comprehensive microbial variant detection and genome assembly improvement. PLOS One 9:e112963. doi:10.1371/journal.pone.0112963 25409509 PMC4237348

[51] Roach MJ, Schmidt SA, Borneman AR. 2018. Purge haplotigs: allelic contig reassignment for third-gen diploid genome assemblies. BMC Bioinformatics 19:460. doi:10.1186/s12859-018-2485-7 30497373 PMC6267036

[52] Morgulis A, Gertz EM, Schäffer AA, Agarwala R. 2006. A fast and symmetric DUST implementation to mask low-complexity DNA sequences. J Comput Biol 13:1028–1040. doi:10.1089/cmb.2006.13.1028 16796549

[53] Stanke M, Keller O, Gunduz I, Hayes A, Waack S, Morgenstern B. 2006. AUGUSTUS: ab initio prediction of alternative transcripts. Nucleic Acids Res 34:W435–W439. doi:10.1093/nar/gkl200 16845043 PMC1538822

[54] Altschul SF, Gish W, Miller W, Myers EW, Lipman DJ. 1990. Basic local alignment search tool. J Mol Biol 215:403–410. doi:10.1016/S0022-2836(05)80360-2 2231712

[55] Eddy SR. 2011. Accelerated profile HMM searches. PLoS Comput Biol 7:e1002195. doi:10.1371/journal.pcbi.1002195 22039361 PMC3197634

[56] Kanehisa M, Goto S. 2000. KEGG: Kyoto encyclopedia of genes and genomes. Nucleic Acids Res 28:27–30. doi:10.1093/nar/28.1.27 10592173 PMC102409

[57] The UniProt Consortium. 2019. UniProt: a worldwide hub of protein knowledge. Nucleic Acids Res 47:D506–D515. doi:10.1093/nar/gky1049 30395287 PMC6323992

[58] Finn RD, Bateman A, Clements J, Coggill P, Eberhardt RY, Eddy SR, Heger A, Hetherington K, Holm L, Mistry J, Sonnhammer ELL, Tate J, Punta M. 2014. Pfam: the protein families database. Nucl Acids Res 42:D222–D230. doi:10.1093/nar/gkt1223 24288371 PMC3965110

[59] Haft DH, Selengut JD, Richter RA, Harkins D, Basu MK, Beck E. 2013. TIGRFAMs and genome properties in 2013. Nucleic Acids Res 41:D387–95. doi:10.1093/nar/gks1234 23197656 PMC3531188

[60] Almagro Armenteros JJ, Tsirigos KD, Sønderby CK, Petersen TN, Winther O, Brunak S, von Heijne G, Nielsen H. 2019. SignalP 5.0 improves signal peptide predictions using deep neural networks. Nat Biotechnol 37:420–423. doi:10.1038/s41587-019-0036-z 30778233

[61] Jones P, Binns D, Chang H-Y, Fraser M, Li W, McAnulla C, McWilliam H, Maslen J, Mitchell A, Nuka G, Pesseat S, Quinn AF, Sangrador-Vegas A, Scheremetjew M, Yong S-Y, Lopez R, Hunter S. 2014. InterProScan 5: genome-scale protein function classification. Bioinformatics 30:1236–1240. doi:10.1093/bioinformatics/btu031 24451626 PMC3998142

[62] Ewels P, Magnusson M, Lundin S, Käller M. 2016. MultiQC: summarize analysis results for multiple tools and samples in a single report. Bioinformatics 32:3047–3048. doi:10.1093/bioinformatics/btw354 27312411 PMC5039924

[63] Chen S, Zhou Y, Chen Y, Gu J. 2018. Fastp: an ultra-fast all-in-one FASTQ preprocessor. Bioinformatics 34:i884–i890. doi:10.1093/bioinformatics/bty560 30423086 PMC6129281

[64] Langmead B, Salzberg SL. 2012. Fast gapped-read alignment with Bowtie 2. Nat Methods 9:357–359. doi:10.1038/nmeth.1923 22388286 PMC3322381

[65] Li H, Handsaker B, Wysoker A, Fennell T, Ruan J, Homer N, Marth G, Abecasis G, Durbin R, 1000 Genome Project Data Processing Subgroup. 2009. The sequence Alignment/Map format and SAMtools. Bioinformatics 25:2078–2079. doi:10.1093/bioinformatics/btp352 19505943 PMC2723002

[66] Liao Y, Smyth GK, Shi W. 2014. featureCounts: an efficient general purpose program for assigning sequence reads to genomic features. Bioinformatics 30:923–930. doi:10.1093/bioinformatics/btt656 24227677

[67] Core Team RD. 2023. R: a language and environment for statistical computing. R Foundation for Statistical Computing, Vienna, Austria.

[68] Love MI, Huber W, Anders S. 2014. Moderated estimation of fold change and dispersion for RNA-seq data with DESeq2. Genome Biol 15:550. doi:10.1186/s13059-014-0550-8 25516281 PMC4302049

[69] Alexa A, Rahnenführer J. 2009. Gene set enrichment analysis with topGO. Bioconductor Improv 27:1–26. 10.18129/B9.bioc.topGO.

[70] Gambarini V, Pantos O, Kingsbury JM, Weaver L, Handley KM, Lear G. 2022. PlasticDB: a database of microorganisms and proteins linked to plastic biodegradation. Database (Oxford) 2022:baac008. doi:10.1093/database/baac008 35266524 PMC9216477

[71] Buchfink B, Xie C, Huson DH. 2015. Fast and sensitive protein alignment using DIAMOND. Nat Methods 12:59–60. doi:10.1038/nmeth.3176 25402007

[72] Jumper J, Evans R, Pritzel A, Green T, Figurnov M, Ronneberger O, Tunyasuvunakool K, Bates R, Žídek A, Potapenko A, et al.. 2021. Highly accurate protein structure prediction with AlphaFold. Nature 596:583–589. doi:10.1038/s41586-021-03819-2 34265844 PMC8371605

[73] Mirdita M, Schütze K, Moriwaki Y, Heo L, Ovchinnikov S, Steinegger M. 2022. ColabFold: making protein folding accessible to all. Nat Methods 19:679–682. doi:10.1038/s41592-022-01488-1 35637307 PMC9184281

[74] Berman HM, Westbrook J, Feng Z, Gilliland G, Bhat TN, Weissig H, Shindyalov IN, Bourne PE. 2000. The protein data bank. Nucleic Acids Res 28:235–242. doi:10.1093/nar/28.1.235 10592235 PMC102472

[75] Velankar S, van Ginkel G, Alhroub Y, Battle GM, Berrisford JM, Conroy MJ, Dana JM, Gore SP, Gutmanas A, Haslam P, et al.. 2016. PDBe: improved accessibility of macromolecular structure data from PDB and EMDB. Nucleic Acids Res 44:D385–95. doi:10.1093/nar/gkv1047 26476444 PMC4702783

[76] Rossum G, Drake FL. 2009. Python 3 reference manual. CreateSpace. doi:10.5555/1593511

[77] Dixon P. 2003. VEGAN, a package of R functions for community ecology. J Vegetation Science 14:927–930. doi:10.1111/j.1654-1103.2003.tb02228.x

[78] Virtanen P, Gommers R, Oliphant TE, Haberland M, Reddy T, Cournapeau D, Burovski E, Peterson P, Weckesser W, Bright J, et al.. 2020. SciPy 1.0: fundamental algorithms for scientific computing in Python. Nat Methods 17:261–272. doi:10.1038/s41592-019-0686-2 32015543 PMC7056644

[79] Seabold S, Perktold J. 2010. statsmodels: econometric and statistical modeling with python. 9th Python in Science Conference; Austin, Texas. doi:10.25080/Majora-92bf1922-011

[80] Gancedo JM. 1998. Yeast carbon catabolite repression. Microbiol Mol Biol Rev 62:334–361. doi:10.1128/MMBR.62.2.334-361.1998 9618445 PMC98918

[81] Simpson-Lavy K, Kupiec M. 2019. Carbon catabolite repression: not only for glucose. Curr Genet 65:1321–1323. doi:10.1007/s00294-019-00996-6 31119370

[82] Ronne H. 1995. Glucose repression in fungi. Trends Genet 11:12–17. doi:10.1016/s0168-9525(00)88980-5 7900189

[83] Lear G, Maday SDM, Gambarini V, Northcott G, Abbel R, Kingsbury JM, Weaver L, Wallbank JA, Pantos O. 2022. Microbial abilities to degrade global environmental plastic polymer waste are overstated. Environ Res Lett 17:043002. doi:10.1088/1748-9326/ac59a7

[84] Sun Z-B, Sun M-H, Li S-D. 2015. Draft genome sequence of mycoparasite Clonostachys rosea strain 67-1. Genome Announc 3:10. doi:10.1128/genomeA.00546-15 PMC4447911 26021926

[85] Broberg M, Dubey M, Sun M-H, Ihrmark K, Schroers H-J, Li S-D, Jensen DF, Brandström Durling M, Karlsson M. 2018. Out in the cold: identification of genomic regions associated with cold tolerance in the biocontrol fungus Clonostachys rosea through genome-wide association mapping. Front Microbiol 9:2844. doi:10.3389/fmicb.2018.02844 30524411 PMC6262169

[86] Astashyn A, Tvedte ES, Sweeney D, Sapojnikov V, Bouk N, Joukov V, Mozes E, Strope PK, Sylla PM, Wagner L, Bidwell SL, Clark K, Davis EW, Smith-White B, Hlavina W, Pruitt KD, Schneider VA, Murphy TD. 2023. Rapid and sensitive detection of genome contamination at scale with FCS-GX. bioRxiv:2023.06.02.543519. doi:10.1101/2023.06.02.543519 PMC10898089 38409096

[87] Nielsen H, Tsirigos KD, Brunak S, von Heijne G. 2019. A brief history of protein sorting prediction. Protein J 38:200–216. doi:10.1007/s10930-019-09838-3 31119599 PMC6589146

[88] Alberts B, Johnson A, Lewis J, Raff M, Roberts K, Walter P. 2015. Molecular biology of the cell. Taylor and Francis group

[89] Houten SM, Wanders RJA. 2010. A general introduction to the biochemistry of mitochondrial fatty acid β-oxidation. J Inherit Metab Dis 33:469–477. doi:10.1007/s10545-010-9061-2 20195903 PMC2950079

[90] Jimenez-Diaz L, Caballero A, Segura A. 2017. Pathways for the degradation of fatty acids in bacteria, p 1–23. In Rojo F (ed), Aerobic utilization of hydrocarbons, oils and lipids. Springer International Publishing, Cham.

[91] Zhang K, Hu J, Yang S, Xu W, Wang Z, Zhuang P, Grossart H-P, Luo Z. 2022. Biodegradation of polyester polyurethane by the marine fungus Cladosporium halotolerans 6UPA1. J Hazard Mater 437:129406. doi:10.1016/j.jhazmat.2022.129406 35753302

[92] Martin F, Canet D, Marchal J-P, Brondeau J. 1984. In vivo natural-abundance C nuclear magnetic resonance studies of living ectomycorrhizal fungi : observation of fatty acids in Cenococcum graniforme and Hebeloma crustuliniforme. Plant Physiol 75:151–153. doi:10.1104/pp.75.1.151 16663561 PMC1066852

[93] Laczko E, Boller T, Wiemken V. 2004. Lipids in roots of Pinus sylvestris seedlings and in mycelia of Pisolithus tinctorius during ectomycorrhiza formation: changes in fatty acid and sterol composition. Plant Cell Environ 27:27–40. doi:10.1046/j.0016-8025.2003.01122.x

[94] Trépanier M, Bécard G, Moutoglis P, Willemot C, Gagné S, Avis TJ, Rioux J-A. 2005. Dependence of arbuscular-mycorrhizal fungi on their plant host for palmitic acid synthesis. Appl Environ Microbiol 71:5341–5347. doi:10.1128/AEM.71.9.5341-5347.2005 16151123 PMC1214663

[95] Ronkvist ÅM, Xie W, Lu W, Gross RA. 2009. Cutinase-catalyzed hydrolysis of poly(ethylene terephthalate). Macromolecules 42:5128–5138. doi:10.1021/ma9005318

[96] Roth C, Wei R, Oeser T, Then J, Föllner C, Zimmermann W, Sträter N. 2014. Structural and functional studies on a thermostable polyethylene terephthalate degrading hydrolase from Thermobifida fusca. Appl Microbiol Biotechnol 98:7815–7823. doi:10.1007/s00253-014-5672-0 24728714

[97] Lai J, Huang H, Lin M, Xu Y, Li X, Sun B. 2022. Enzyme catalyzes ester bond synthesis and hydrolysis: The key step for sustainable usage of plastics. Front Microbiol 13:1113705. doi:10.3389/fmicb.2022.1113705 36713200 PMC9878459

[98] Di Bisceglie F, Quartinello F, Vielnascher R, Guebitz GM, Pellis A. 2022. Cutinase-catalyzed polyester-polyurethane degradation: elucidation of the hydrolysis mechanism. Polymers (Basel) 14:411. doi:10.3390/polym14030411 35160402 PMC8838978

[99] Vázquez-Alcántara L, Oliart-Ros RM, García-Bórquez A, Peña-Montes C. 2021. Expression of a cutinase of Moniliophthora roreri with polyester and PET-plastic residues degradation activity. Microbiol Spectr 9:e0097621. doi:10.1128/Spectrum.00976-21 34730414 PMC8567236

[100] Miyakawa T, Mizushima H, Ohtsuka J, Oda M, Kawai F, Tanokura M. 2015. Structural basis for the Ca(2+)-enhanced thermostability and activity of PET-degrading cutinase-like enzyme from Saccharomonospora viridis AHK190. Appl Microbiol Biotechnol 99:4297–4307. doi:10.1007/s00253-014-6272-8 25492421

[101] Hisano T, Kasuya K-I, Tezuka Y, Ishii N, Kobayashi T, Shiraki M, Oroudjev E, Hansma H, Iwata T, Doi Y, Saito T, Miki K. 2006. The crystal structure of polyhydroxybutyrate depolymerase from Penicillium funiculosum provides insights into the recognition and degradation of biopolyesters. J Mol Biol 356:993–1004. doi:10.1016/j.jmb.2005.12.028 16405909

