# Supplementary material for: Molecular mechanisms of plastic biodegradation by the fungus Clonostachys rosea
Source: mBio. 2025 Jun 30;16(8):e00335-25. doi: 10.1128/mbio.00335-25 (PMC12345228; doi:10.1128/mbio.00335-25)
Supplement: Supplemental figures and tables — Figures S1 to S7; Tables S1 and S2. [file mbio.00335-25-s0001.docx]

**Molecular mechanisms of plastic biodegradation by the fungus *Clonostachys rosea***

Victor Gambarini^1*^, Nikolai Pavlov^1^, Paul Young^1^, Stephanie Dawes^1^, Arnaud Auffret^1^, Joanne M. Kingsbury^2^, Lloyd A. Donaldson^3^, Dawn A. Smith^3^, Louise Weaver^2^, Olga Pantos^2^, Kim M. Handley^1^, Gavin Lear^1^

^1^School of Biological Sciences, University of Auckland, 3a Symonds Street, Auckland 1010, New Zealand.

^2^The Institute of Environmental Science and Research, 27 Creyke Road, Ilam, Christchurch 8041, New Zealand.

^3^Scion, Te Papa Tipu Innovation Park, Rotorua, New Zealand.

^*^Corresponding author.

victor.gambarini@auckland.ac.nz


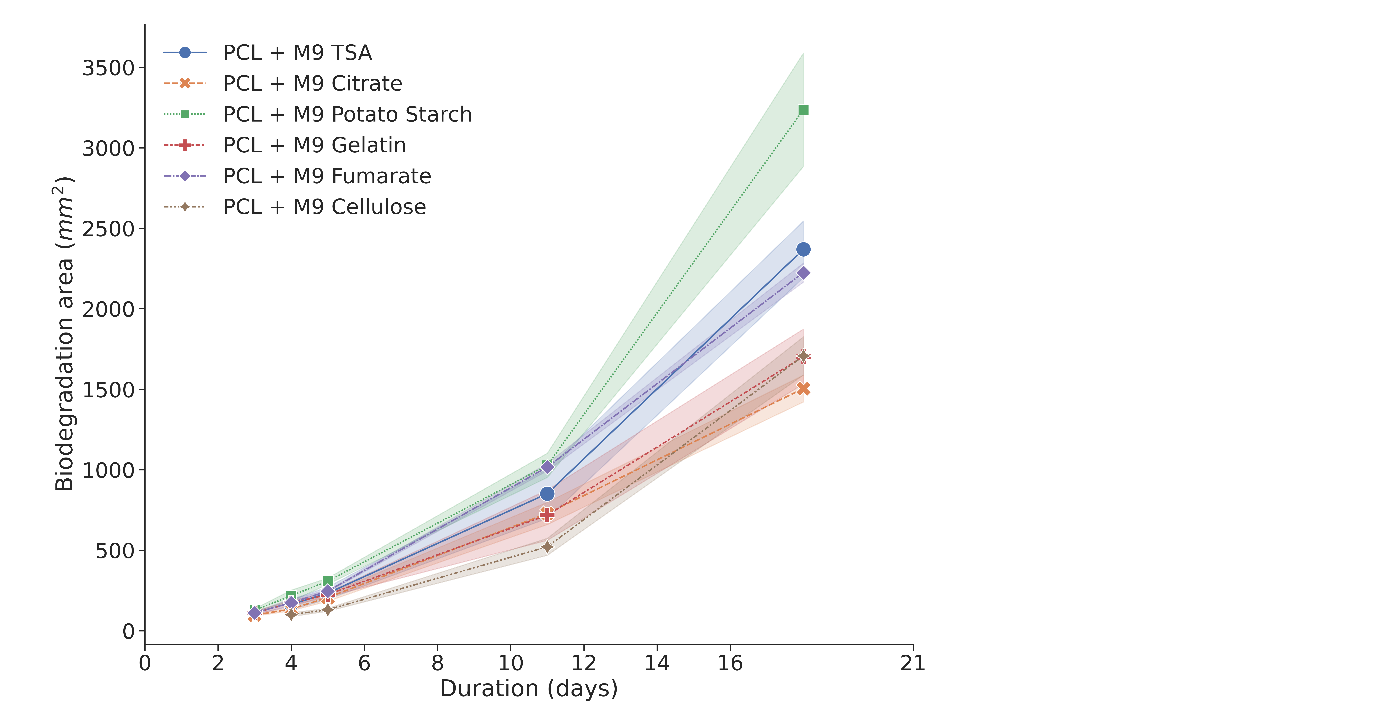


**Supplementary Figure 1**: Effect of different carbon sources on PCL biodegradation by *Clonostachys rosea*. The ability of *C. rosea* to degrade PCL emulsion was assessed in M9 media supplemented with various carbon sources (TSA, citrate, potato starch, gelatin, fumarate, and cellulose). Biodegradation was quantified by measuring the area of the clear halo formed around the microbial colonies over 18 days. Each treatment was performed in triplicate. Shaded error bands represent standard deviation values. Statistical analysis (ANOVA) on day 18 revealed significant differences among media types (F = 158.51, p < 0.001). Tukey's HSD post hoc test identified pairwise variations, except for cellulose-(citrate, gelatin), citrate-gelatin, and fumarate-TSA comparisons.

**Supplementary Table 1**: Quality control of reads produced for the whole genome of *Clonostachys rosea*. The functions ‘Trimmomatic’ and ‘Sickle’ remove primer/adaptor and low-quality DNA sequences, respectively.

| **Step** | **Statistic** | **Value** |
| --- | --- | --- |
| Initial | Number of paired reads | 8,524,227 |
| Trimmomatic | Number of paired reads removed | 5,140 |
|  | Number of paired reads removed (%) | 0.06% |
| Sickle | Number of paired reads removed | 101,183 |
|  | Number of paired reads removed (%) | 1.19% |
| Final | Number of paired reads kept | 8,417,904 |
|  | Number of paired reads kept (%) | 98.75% |

**Supplementary Table 2**: Genome assembly statistics for *Clonostachys rosea,* produced by SPAdes for contigs >= 500 bp, unless otherwise noted (e.g., "# contigs (>= 0 bp)" and "Total length (>= 0 bp)" include all contigs). More details on genome assembly metrics were published by Thrash *et al.* (2020).

| **Metric** | **Value** |
| --- | --- |
| # contigs (>= 0 bp) | 513 |
| # contigs (>= 1000 bp) | 320 |
| # contigs (>= 5000 bp) | 297 |
| # contigs (>= 10000 bp) | 279 |
| # contigs (>= 25000 bp) | 238 |
| # contigs (>= 50000 bp) | 191 |
| Total length (>= 0 bp) | 56,791,467 |
| Total length (>= 1000 bp) | 56,732,772 |
| Total length (>= 5000 bp) | 56,682,167 |
| Total length (>= 10000 bp) | 56,556,336 |
| Total length (>= 25000 bp) | 55,855,219 |
| Total length (>= 50000 bp) | 54,171,614 |
| # contigs (>= 500 bp) | 351 |
| Largest contig (bp) | 1,262,786 |
| Total length (bp) | 56,752,586 |
| GC (%) | 49.48 |
| N50 | 378,500 |
| N75 | 204,792 |
| L50 | 40 |
| L75 | 91 |
| # N's per 100 kbp | 3.51 |


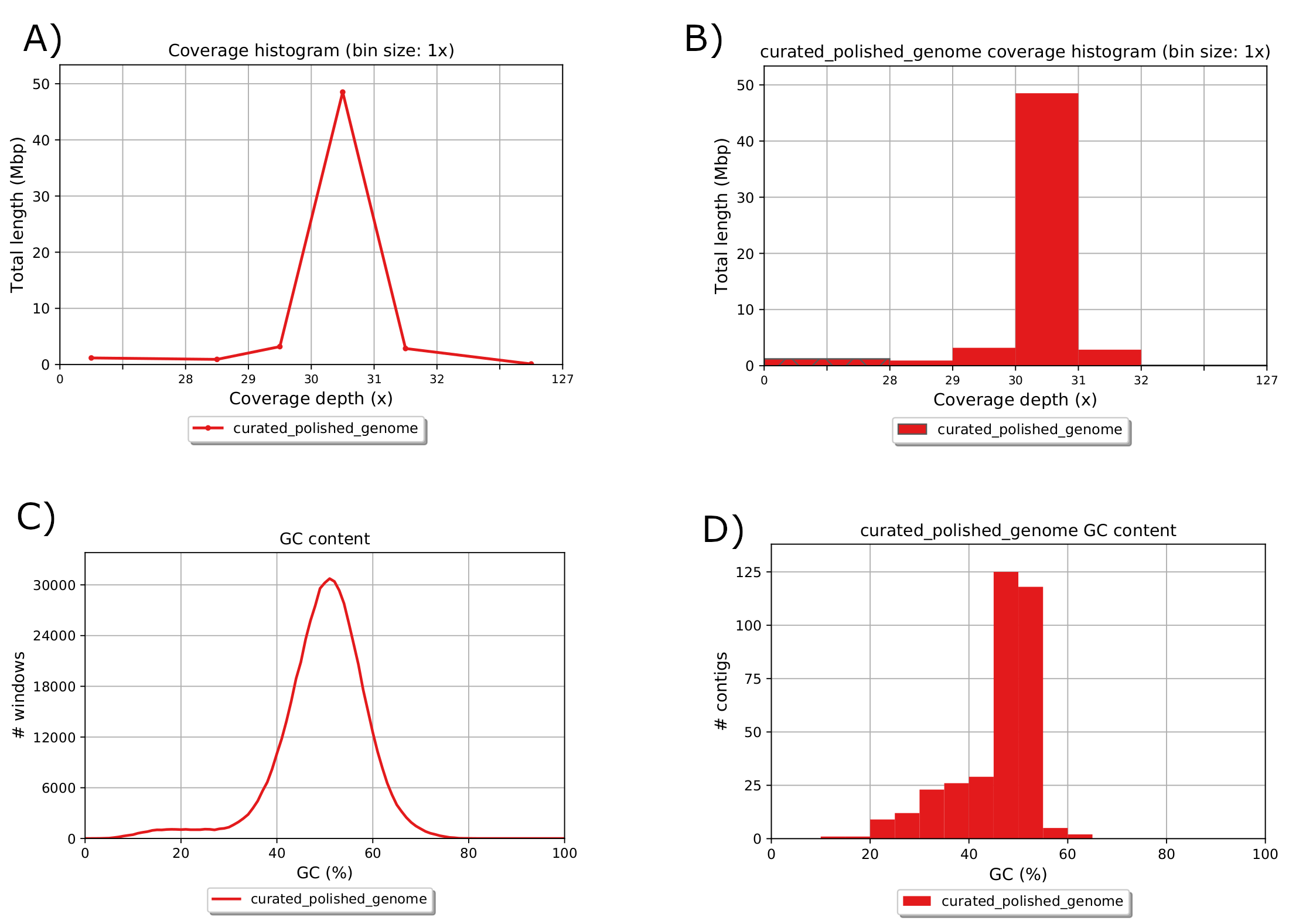


**Supplementary Figure 2**: Genome assembly statistics generated using QUAST software.


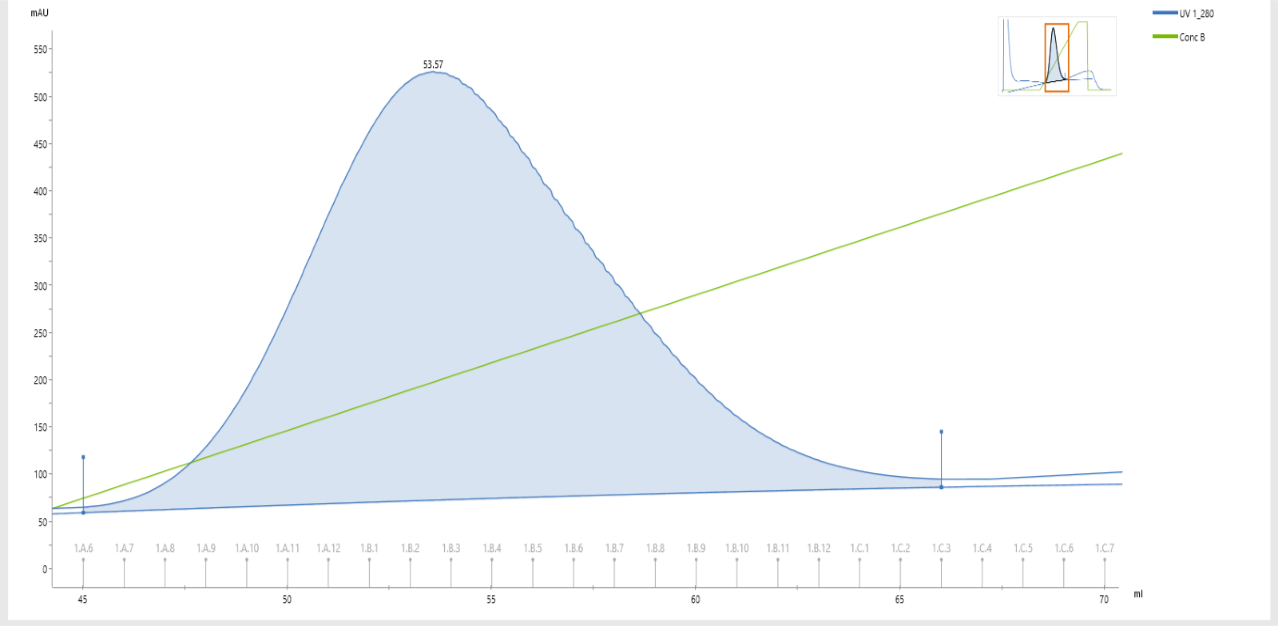


**Supplementary Figure 3**: HPLC peak of protein p9562 (PLDB00213).


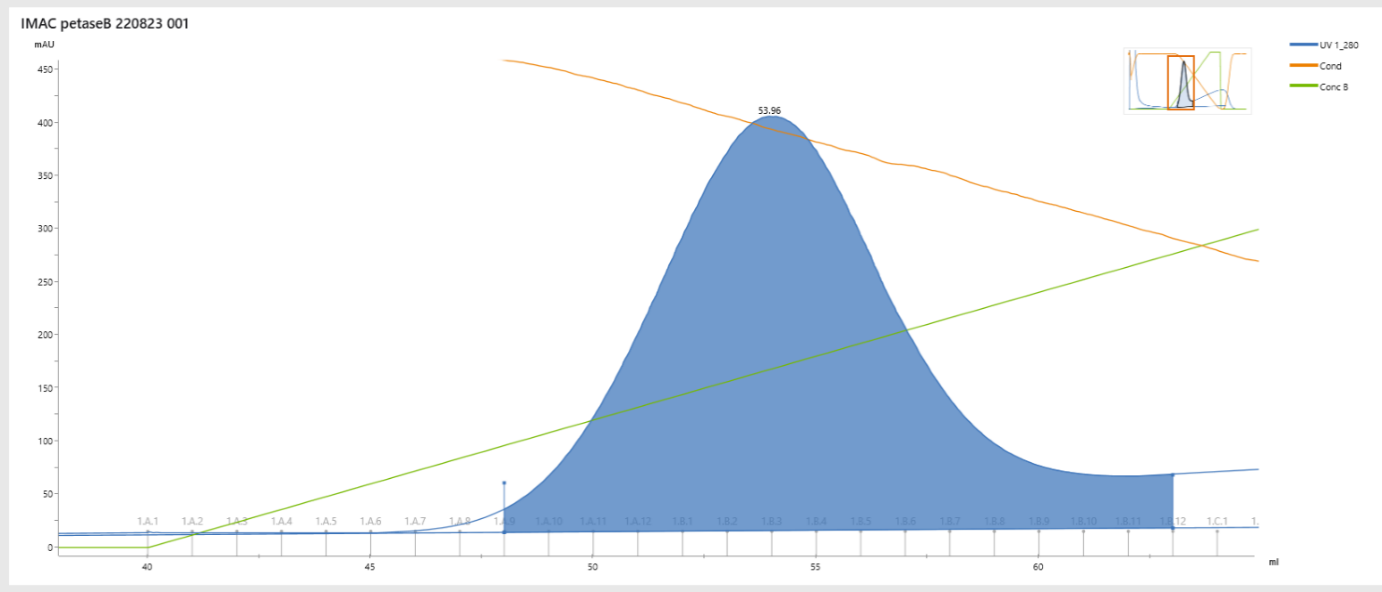


**Supplementary Figure 4**: HPLC peak of protein p16887 (PLDB00214).


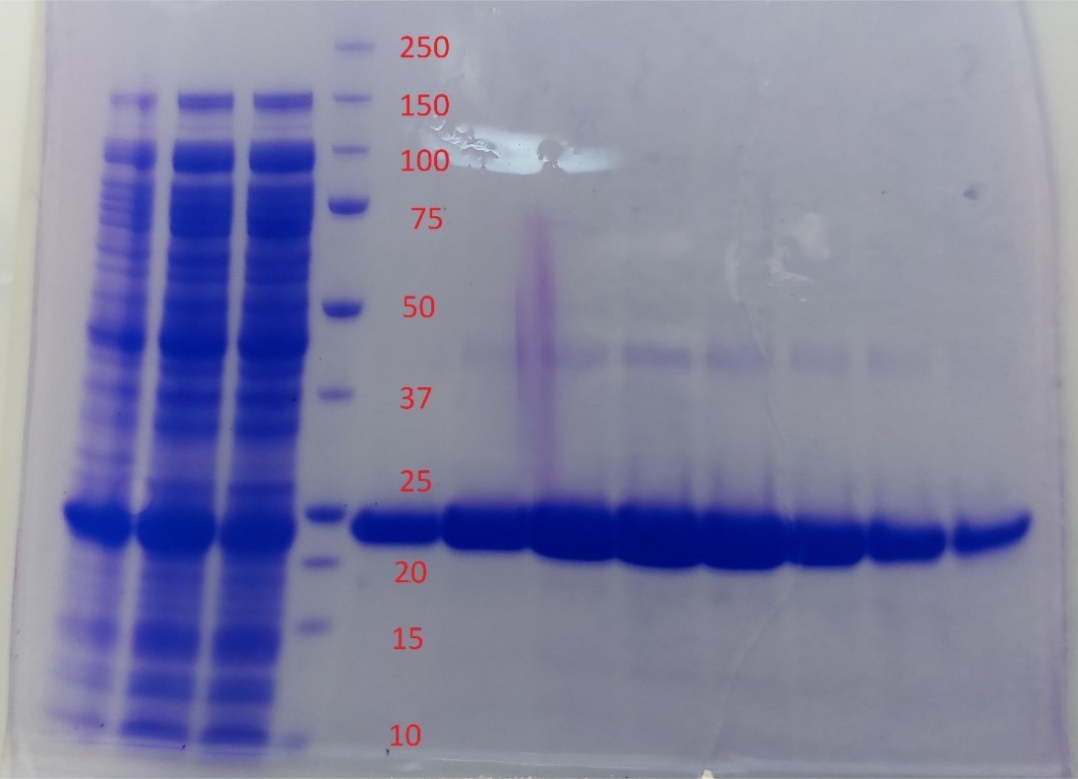


**Supplementary Figure 5**: SDS-PAGE of protein p9562 (PLDB00213).


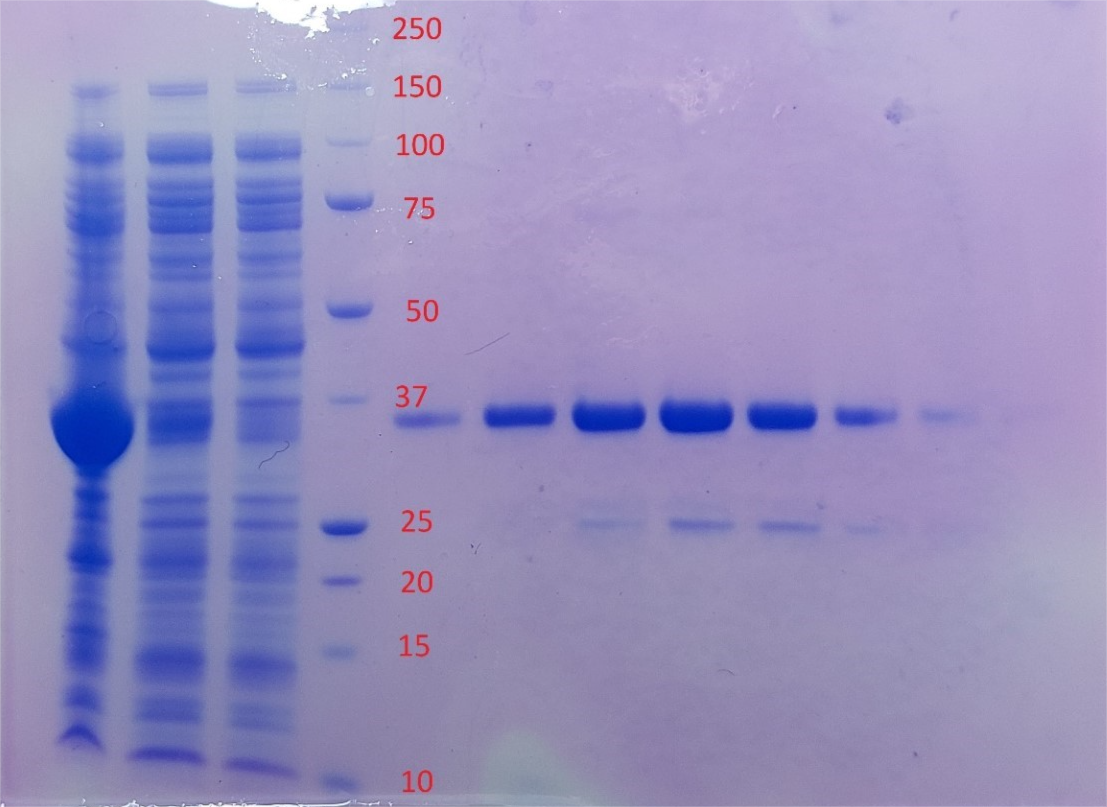

**Supplementary Figure 6**: SDS-PAGE of protein p16887 (PLDB00214).


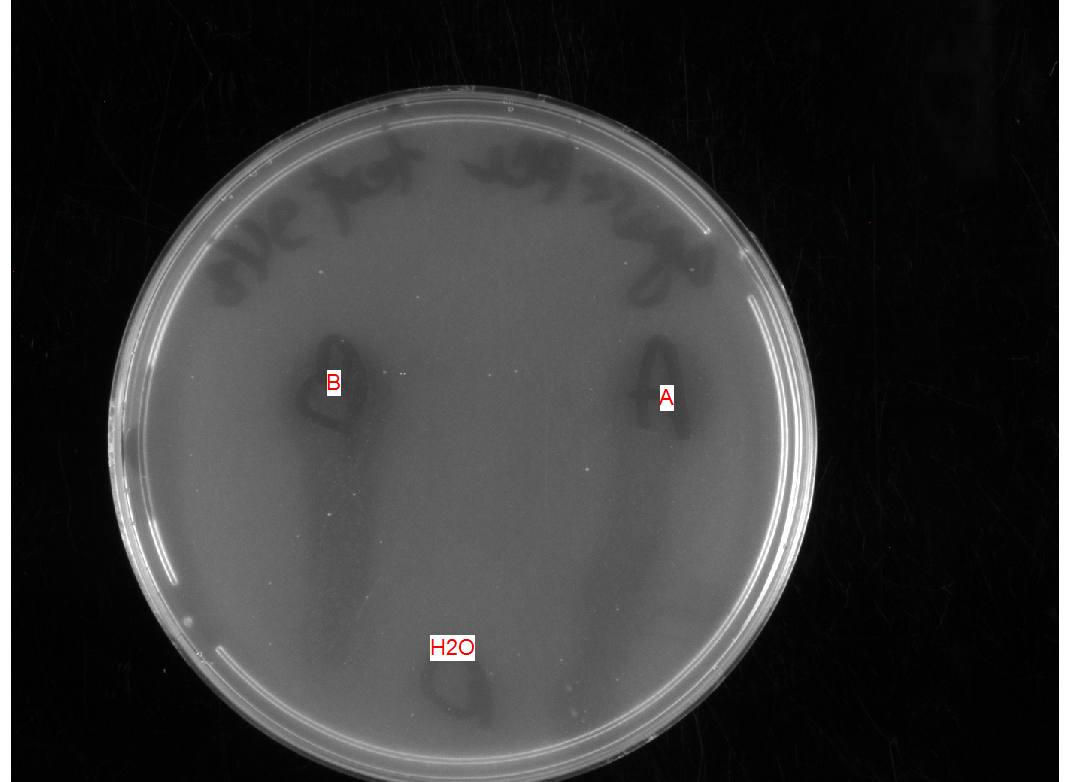


**Supplementary Figure 7**: PCL degradation assay on 0.08% emulsified PCL agar plate. The plate was incubated for five days at room temperature. A: enzyme PLDB00213. B: enzyme PLDB00214. H2O: water.

# REFERENCES

Thrash A, Hoffmann F & Perkins A (2020) Toward a more holistic method of genome assembly assessment. *BMC Bioinformatics* 21: 249.
